# A Pilot Standardized Simulation-Based Mechanical Ventilation Curriculum Targeting Pulmonary and Critical Care Medicine and Critical Care Medicine Fellows

**DOI:** 10.1055/s-0043-1773792

**Published:** 2023-10-03

**Authors:** Amina Pervaiz, Asil Daoud, Abdulrazak Alchakaki, Shyam Ganti, Divya Venkat, Sarah Lee, Abdulghani Sankari

**Affiliations:** 1 Division of Pulmonary and Critical Care, Detroit Medical Center - Wayne State University School of Medicine, Detroit, Michigan, United States; 2 Division of Pulmonary and Critical Care, John D. Dingell VA Medical Center, Detroit, Michigan, United States; 3 Department of Pulmonary, Critical Care and Sleep Medicine, William Beaumont Hospital, Royal Oak, Michigan, United States; 4 Division of Pulmonary and Critical Care, University of Maryland Medical Center, Baltimore, Maryland, United States; 5 Department of Pulmonary, Critical Care and Sleep Medicine, Appalachian Regional Healthcare Hospital, Harlan, Kentucky, United States; 6 Department of Education, John D. Dingell VA Medical Center, Detroit, Michigan, United States; 7 Division of Pulmonary and Critical Care, Cleveland Clinic, Cleveland, Ohio, United States; 8 Department of Medical Education, Ascension Providence Hospital, Southfield, Michigan, United States

**Keywords:** simulation, mechanical ventilation, fellowship, competency

## Abstract

**Introduction** The mastery of mechanical ventilation (MV) management is challenging, as it requires the integration of physiological and technological knowledge with critical thinking. Our aim was to create a standardized curriculum with assessment tools based on evidence-based practices to identify the skill deficit and improve knowledge in MV management.

**Methods** For 3 years, 3 hours of standardized curriculum for each first-year pulmonary critical care medicine (PCCM) and critical care medicine (CCM) fellows was integrated into the orientation (chronologically): (1) a baseline knowledge pretest; (2) a 1-hour one-on-one case-based simulation session with debriefing. A 34-item competency checklist was used to assess critically thinking and skills and guide the debriefing; (3) a 1-hour group didactic on respiratory mechanics and physiology; (4) a 45-minute hands-on session in small groups of one to three fellows for basic knobology, waveforms, and various modes of mechanical ventilators; (5) a 15-minute group bedside teaching of vented patients covering topics such as techniques to alleviate dyssynchrony and advanced ventilator modes; (6) a one-on-one simulation reassessment session; (7) a knowledge posttest. Fellows' performances at baseline, 1-month posttest, and end-of-first year post-test were compared.

**Results** Fellows ( *n* = 24) demonstrated significant improvement at 1-month posttest in knowledge (54.2% ± 11.0 vs. 76.6 ± 11.7%, *p* < 0.001) and MV competency (40.7 ± 11.0% vs. 69.7 ± 9.3%, *p* < 0.001), compared with pretest. These improvements were retained at the end-of-year reassessments (knowledge 75.1 ± 14.5% and MV competency 85.5 ± 8.7%; *p* < 0.001).

**Conclusion** Standardized simulation-based MV curriculum may improve the medical knowledge competency, and confidence of first-year PCCM and CCM fellows toward MV management before encountering actual ventilated patients.

## Introduction

Mechanical ventilation (MV) management is an essential skill typically acquired through experience and repeated exposure to intubated and mechanically ventilated patients during the medical training; however, there is no consensus on the best approach to educate this core competency. 1 2 3 4 5 One of the major teaching challenges is due to the fact that diagnosis and treatment of acute respiratory failure with advanced airway and ventilation strategies entail a high learning curve to understanding complex pathophysiology and technology. 6 7 This can lead to major knowledge gaps among trainees, including important learning objectives such as optimizing ventilator modes and weaning strategies for good patient outcome. 8 9 Detecting and addressing gaps in knowledge and skill early in training is paramount for patient safety. 10 In addition, acquiring theoretical knowledge through educational models, such as reading and attending lectures, may not translate to appropriately applying evidence-based guidelines and protocols to clinical practice. 4 8 11 12 13 Simulation-Based Medical Education (SBME) is well suited for assessing knowledge, critical thinking skills, and confidence while allowing sufficient time for the educator to probe learners' thought processes and cognitive biases, as well as the ability to tailor one-on-one teaching to address gaps without compromising patient safety. 4 9 13 14 15 Using a high-fidelity MV simulation for graduate medical education has been shown to improve learner outcomes over other types of teaching; however, those studies have focused at an introductory level or a specific aspect of ventilation but not a full-spectrum curriculum for advanced learners. 13 16 17 18 Therefore, we aimed to develop a comprehensive high-fidelity case-based simulation curriculum and assessment checklist rooted in evidence-based practices for MV management. This curriculum will assess incoming pulmonary critical care medicine (PCCM) and critical care medicine (CCM) fellows' medical knowledge and cognitive skills toward MV management at the beginning of their fellowship prior to starting clinical rotations. Preliminary results of this simulation-based curriculum were previously reported in abstract form and presented at the 2018 international convention of the American Thoracic Society. 19

## Methods

### Development

This MV simulation curriculum was designed as an educational tool to improve the medical knowledge and skill competency in MV management of first-year PCCM and CCM fellows. This course was conducted for three subsequent years, and the results were pooled together for analysis. Incoming first-year physicians-in-training in PCCM and CCM fellowships (referred to as “learners” later) at a single large tertiary teaching institution participated during a formal orientation or “boot camp” during the July months of 2017, 2018, and 2019, before starting any rotations in ICUs. This study was declared exempt by the Wayne State University (WSU) Institutional Review Board Administration Office.

### Assessment

A multiple-choice questionnaire (MCQ) and competency checklist were created to assess pre- and post-curriculum knowledge and skills competencies. The 15 MCQs to assess cognitive skills and medical knowledge were created based on CHEST SEEK questions (Critical Care Medicine: 26th edition) and modified by the two faculty members of the MV training team.

A simulation case scenario was used for critical thinking and skills assessment ( Supplementary Appendix A , available online). The case scenario begins with a patient with a history of asthma presenting to the emergency department with acute respiratory failure, then rapidly deteriorating, requiring initiation of MV. The learner has to decide to intubate and optimize immediate post-intubation care. The scenario continues through the hospitalization complicated with auto-PEEP development, mucous plug, ARDS, and various ventilator asynchronies, prompting learners to recognize issues and make appropriate adjustments to the MV management. The last phase of the scenario requires the learner to assess readiness to liberate from the ventilator and decide on transferring out of the ICU. Based on needs assessment survey of teaching faculty, the following topics were identified as essential for improvement among our institution's fellows: medical decision-making between the use of noninvasive positive pressure ventilators versus invasive mechanical ventilator, use of different ventilator modes and settings, immediate postintubation care including ventilator complication prophylaxis or “ventilator bundles,” interpreting the ventilator-generated data (especially waveforms depicting dynamic hyperinflation and ventilator asynchrony), analyzing elevated peak versus plateau pressures, ARDS management, and ventilator weaning. Using this framework, we designed five unique lung models on the ASL 5000 simulator to mimic the ventilator physiology of differing respiratory disease processes (normal lungs, dynamic hyperinflation, elevated airway resistance, noncompliant lung, and lung with flow asynchrony) and a 34-item competency checklist ( Supplementary Appendix B , available online). The learner is expected to think aloud, make medical decisions, and react in real time to the consequences of these decisions, as demonstrated in a high-fidelity simulation. Competency items were decided and approved by two board-certified CCM faculty based on evidence-based practices of MV, including indications, initiation, troubleshooting, and liberation of MV. 7 8 9 The proctor's prompts in the case scenario were scripted (with open-ended questions) to avoid leading questions or bias toward the trainee. Points were given on the 34-item competency checklist based on the grading guidelines ( Supplementary Appendix E , available online).

### Equipment

The simulation scenario was conducted in a controlled environment in the simulation laboratory. Materials and equipment used included the following: a high-fidelity Laerdal manikin lying on a stretcher or adjustable bed with the capability to demonstrate clinical exam findings such as breath and heart sounds, ASL 5000 Breathing Simulator by Ingmar Medical connected to a computer programmed with the different lung models, a monitor to display dynamically changing generated vital signs, a mechanical ventilator to demonstrate waveforms and settings, and another monitor to display imaging and information relevant to the case scenario. Equipment and environment were designed to create an authentic in situ experience as much as possible by using real equipment (i.e., the actual mechanical ventilator used in the hospital) and supplies (i.e., endotracheal tube; placed in the manikin when the patient was intubated in the scenario), non-rebreather mask connected to wall oxygen, a feeding tube (placed when the learner asked for it), venturi mask and nasal cannula (on extubation), empty 10-mL syringe with Luer lock (for endotracheal tube cuff inflation/deflation), and bag-valve mask.

### Personnel

In 2017, all the assessment tools and learning materials were developed for this curriculum by a dedicated five-member MV team. This team comprised two teaching faculty physicians in the Division of Pulmonary, Critical Care, and Sleep Medicine with educational and test writing expertise where both were simulation directors at different centers, one senior PCCM fellow designated in the clinician-educator (CE) track, a former dean of education, and a respiratory therapist (RT). Initially, in July 2017, every simulation testing session had a four-member team, each performing predefined roles: one team member acted as a proctor prompting the learner with scripted open-ended questions at each scenario's branch point based on a predetermined decision tree; another made changes on the lung simulator and mechanical ventilator, and two unblinded members observed and marked the competency checklist (see Supplementary Appendix C , available online). After 2018, efficiency was improved such that only two team members were needed for every simulation session with one learner. One member, the CE track fellow, was able to proctor the session, make changes to the lung simulator and mechanical ventilator, and score the trainee's competency checklist, while the second member, a teaching faculty board-certified in CCM, silently scored a second competency checklist and contributed to the debriefing and teaching. All team members adhered to a grading criterion and were assigned to learners to keep the consistency of the assessors throughout the baseline and follow-up testing (see grading guidelines in Supplementary Appendix).

### Debriefing

Immediately following every simulation session was a debriefing session, which was structured around the learner's self-reflection and the competency checklist to review the learner's adherence to evidence-based best practices. The actual material used during debriefing is attached in Supplementary Appendix C , available online. Time spent on each topic was individualized to the learner's performance or needs to fully address questions about any segment of the case scenario.

### Implementation

Trainees' baseline and end-of-curriculum performances were measured and compared using knowledge, competency, and satisfaction assessment tools ( Supplementary Appendices A, B , available online) that were developed for this course. Baseline assessment consisted of a written knowledge pretest and demonstration of skills competency during a simulation session. The simulation sessions were immediately followed by one-on-one teaching during the debriefing ( Supplementary Appendix C , available online). In addition to the pretest simulation debriefing, training included a group interactive didactic presentation and two small group workshops, one on ventilator knobology and another on “mini” bedside ICU round focusing solely on ventilator management ( Supplementary Appendix D , available online). Before advancing to their second year in fellowship training, learners underwent an end-of-year knowledge and competency retention assessment ( Supplementary Appendices A, B , available online). The end-of-year retention was performed at an average of 9 months into the first year of fellowship and timed after learners completed two ICU rotations to maintain uniformity of their ICU experience for comparison.

Results were reported using mean and standard deviation (SD). Matched paired *t* -test analysis was conducted with the trainees' pretest scores considered as their baseline control. For statistical analysis, SigmaStat was used (version 3.5; San Jose, California, United States).

## Results

### Knowledge Assessment

**Fig. 1:**
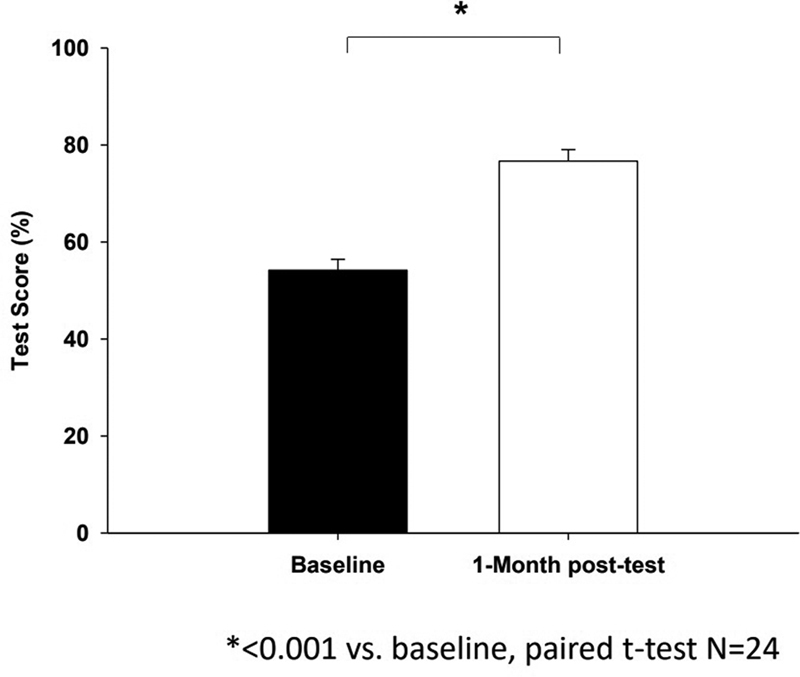
A summary of average mechanical ventilation knowledge test scores at baseline and 1-month posttest ( *N* = 24). * *p* < 0.001 versus baseline using paired *t* -test. Significant improvement in the mean knowledge test score of trainees on y-axis, from 54.2 ± 11.0% at baseline (x-axis) to 76.7 ± 11.6% ( *p* <0.001) at the 1-month posttest on x-axis.

Between 2017 and 2019, a total of 24 trainees participated in the MV course as part of their orientation (PCCM = 18, CCM = 6). Trainees demonstrated significant improvement in the mean knowledge test score, from 54.2 ± 11.0% at baseline to 76.7 ± 11.6% ( *p* < 0.001) at the 1-month post-test ( Fig. 1 ).

### Competency Assessment

**Fig. 2:**
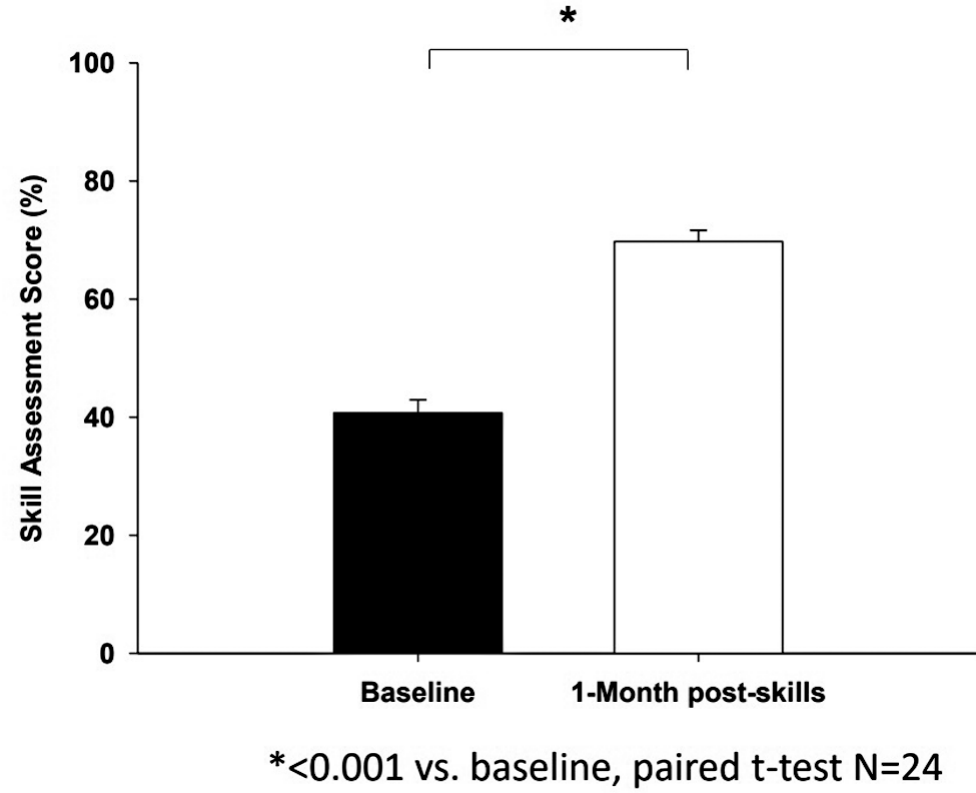
A summary of the average of mechanical ventilation competencies scores at baseline and 1-month posttest ( *N* = 24). * *p* < 0.001 versus baseline using paired *t* -test. Skill assessment on MV competency checklist (y-axis) showed significant improvement from 40.7 ± 11.0% (13.8/34 items) at baseline (x-axis) to 69.7 ± 9.3% (23.7/34 items) at the 1-month posttest, p < 0.001 (x-axis).

The average number of completed MV competency items on the checklist during the simulation testing session showed a significant improvement from 40.7 ± 11.0% (13.8/34 items) at baseline to 69.7 ± 9.3% (23.7/34 items) at the 1-month post-test, *p* < 0.001 ( Fig. 2 ).

### Retention Assessment

**Fig. 3:**
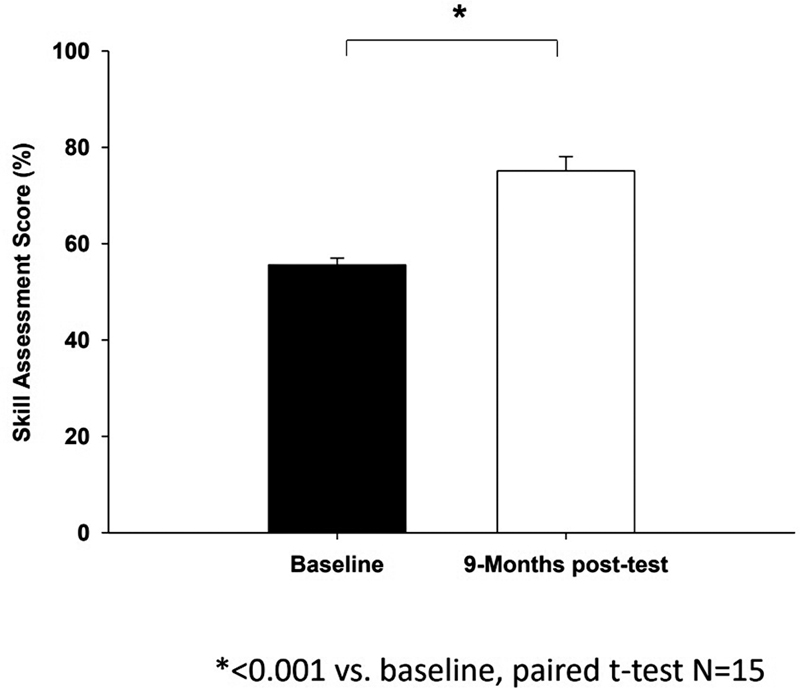
A summary of mean medical knowledge test scores at baseline and end-of-course, respectively ( *N* = 15). * *p* < 0.001 versus baseline using paired *t* -test. Skill assessment on the mean medical knowledge test scores (y-axis) at the end-of-curriculum retention assessment (75.1 ± 14.5%) showed significant improvement from baseline (p < 0.001) on x-axis.

A total of 15 trainees out of 24 completed end-of-year retention assessment sessions. Nine trainees were unable to participate in the end-of-year retention assessment due to the COVID-19 pandemic, disrupting all simulation testing due to the need for all faculty and trainees to focus on treating patients. The mean medical knowledge test scores at the end-of-curriculum retention assessment (75.1 ± 14.5%) showed significant improvement from baseline ( *p* < 0.001), as shown in Fig. 3 .

**Fig. 4:**
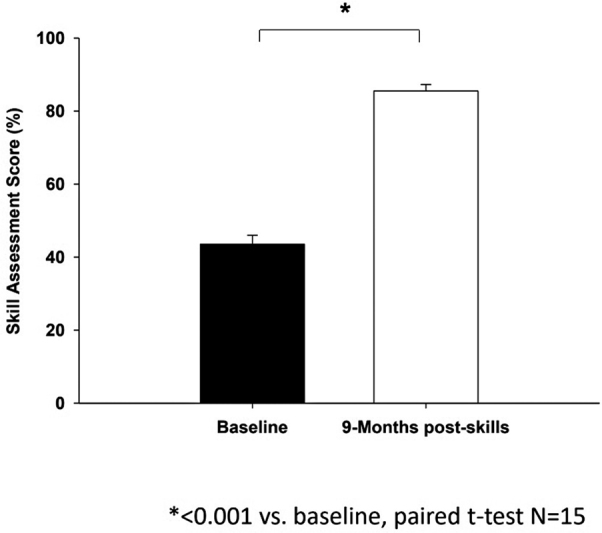
A summary of mean mechanical ventilation competencies scores at baseline and end-of-course, respectively ( *N* = 15). * *p* < 0.001 versus baseline using a paired *t* -test. The MV competency item score on *y* -axis (85.5 ± 8.7%, 29.1/34 items) at the end-of-curriculum retention assessment showed significant improvement from baseline ( *p* < 0.001), as shown in Fig. 4 .

The MV competency item score (85.5 ± 8.7%, 29.1/34 items) at the end-of-curriculum retention assessment showed significant improvement from baseline ( *p* < 0.001), as shown in Fig. 4 .

### Satisfaction Assessment

The curriculum was highly rated by trainees with a mean satisfaction score of 4.7 on a 5-point Likert scale, with 1 being least satisfied and 5 being highly satisfied. Trainees perceived the curriculum as practical and interactive.

## Discussion

### Summary of Findings

Our study demonstrated the following novel findings: (1) Administration of a simulation-based curriculum for incoming PCCM and CCM fellows allowed for the evaluation of knowledge, critical thinking, and skills in MV. (2) The new MV curriculum was associated with statistically significant medical knowledge and skills sustained improvement in the first fellowship year. (3) The curriculum was associated with high satisfaction rates among new PCCM and CCM fellows. In a prospective, randomized cluster study, Schroedl et al compared simulation-based training with traditional training on first-year residents before their first ICU clinical rotation month with MV as one subject among others such as circulatory shock. 4 Their study showed that the simulation-trained group scored significantly higher on a 14-item checklist than the control traditional-trained group and that skills learned during the simulation sessions were transferrable to the bedside practice and improved residents' satisfaction. One study found no difference in knowledge acquisition on ventilator management between using a computer case-based simulation versus a high-fidelity manikin simulation among nurse practitioners. 12 Another study compared computer simulation versus live animal models to teach MV management concepts and found no difference in a 12-question knowledge quiz. 13 Only one other study with PCCM and CCM fellowship trainees using MV simulation has been published, where knowledge test scores after a hands-on tutoring workshop compared with a self-guided learning program that consisted of online modules and selected reading materials did not reach statistical significance, albeit the simulation workshop provided greater learner satisfaction. 3 In contrast, due to our course's learning objectives for comprehensive respiratory failure and MV management, the incorporation of a high-fidelity simulation allowed assessment of cognitive and skill competencies including real-time critical thinking during a simulated crisis and effective communication with MV team members in the room. Similarly, another study for anesthesia residents found a manikin-based simulation more effective than a computer-based one for skill assessments. 10

Strengths of the curriculum include a standardized format to decrease assessment bias, multimodal to address varied learning styles, and adaptability for individualized 1:1 teaching through simulation debriefing and hands-on bedside teaching. PCCM and CCM fellows' MV medical knowledge scores and MV competency significantly improved at the 1-month posttest and maintained by the end of their first academic year without the knowledge or skill decay. The curriculum was also well perceived by trainees with high satisfaction scores based on a 5-point Likert scale. Our study is also unique from previously published studies in that we examined the retention of skills and knowledge on MV almost 1 year later after the initial baseline training. Through the postsimulation debriefing sessions, instruction and guidance were highly individualized with open individualized interaction between learner and instructors to address specific learner's skill or knowledge gaps. To maintain consistency and minimize operator bias in grading, two instructors independently scored the competency checklist for each learner session, and both contributed to the debriefing. Some studies have used simulation training in MV to improve specific skills and knowledge with a pretest and posttest, but only as an introductory course. 1 3 16 20 21 To the best of our knowledge, this will be the first published multimodal simulation-based curriculum aimed at deep learning of MV for PCCM and CCM fellows over their first year in training. The strength of our curriculum is objective medical knowledge assessments, didactic and hands-on lectures, individual simulation sessions with competency skill assessments that included debriefing, bedside rounds, retention reassessments, and trainees' satisfaction surveys.

However, several limitations may influence the interpretation of the results in this study. First, a small sample size at a single institution necessitated evaluation over a period of three consecutive years (2017–2019) and insufficient group size for a control or comparative group. Second, this study may have referral bias and experimenter expectancy, as some of the instructors for this course were also on the course development team. Third, the costs and logistics of implementing a similar simulation-based course may be a resource-limiting step for most institutions. Fourth, learners were tested only in the simulation environment limiting the ability to assess the direct impact on patient outcomes. Fifth, acquiescence bias or maturation effect is a confounding factor since learners' improvement as compared with their baseline rather than a randomized control group who did not undergo this course. It is unknown if the equivalent 1-month posttest and end-of-the-year improvements could be achieved with experiential learning alone. However, this is unlikely because, at the 1-month posttest, the learners did not yet gain significant ICU experience, varying from no ICU exposure to a maximum of one to two 12-hour ICU shifts during this timeframe due to the nature of our fellowship orientation bootcamp, which is dedicated to educational activities rather than clinical assignments. Sixth, test–retest bias is possible since the same test questions and case scenarios were used. This was minimized with the 1-month posttest washout period, immediately collecting all completed knowledge tests, not providing the tests' answers, and mixing the posttest questions in a different sequential order. Seventh is the bias of rating the training by the trainees in the same program. Finally, due to the COVID-pandemic and the need for all to be deployed to work in the ICU, simulation-based testing was disrupted; so end-of-year retention assessment was not captured for nine trainees.

Future directions for this curriculum include improving it to be scaled to other institutions using interactive computerized techniques for simulated learning, which will allow feedback on a large scale from learners and flexibility of adjusting the material in real time. In addition, future studies could benefit from utilizing the competency checklist at the bedside with structured MV rounds to evaluate if performance in a simulation would translate to effective bedside clinical performance by the trainees (change in behavior) and ultimately assess the impact of this education program on clinical outcomes.

In conclusion, we present a novel standardized simulation curriculum that includes evaluation tools for knowledge, critical thinking, and skills for mechanical ventilator management.
