# Supplementary material for: A Pilot Standardized Simulation-Based Mechanical Ventilation Curriculum Targeting Pulmonary and Critical Care Medicine and Critical Care Medicine Fellows
Source: Avicenna J Med. 2023 Oct 3;13(3):176–81. doi: 10.1055/s-0043-1773792 (PMC10550363; doi:10.1055/s-0043-1773792)
Supplement: Supplementary file 1 — Supplementary Appendix A [file 10-1055-s-0043-1773792-s236a.pdf]

Supplementary Appendix List

A. Mechanical ventilator simulation clinical case scenario, Instructor notes—changes and case branch points.
B. Multiple-choice questions for cognitive assessment, critical action checklist for mechanical ventilation competency, and satisfaction assessment.
C. Case simulation and debriefing materials.
D. Implementation information.
E. Grading guidelines for the MV competency checklist.

Supplementary Appendix A Simulation case template

SIMULATION CASE TITLE: Mechanical Ventilator Simulation—Clinical Case Scenario<br>AUTHORS: Abdulrazak Alchakaki, MD,[1,2,6] Amina Pervaiz, MD,[1] Sammar Alsunaid, MD,[1,3] Navin Durairajan, MD,[1] Shyam Ganti, MD,[1,4] Maryjean Schenk, MD, MPH, MS,[5] Divya Venkat, MD,[6] Sarah Lee, MD, MPH,[1,5,6] Abdulghani Sankari, MD, PhD[1,6,8]<br>LEARNER AUDIENCE: First-Year Pulmonary and Critical Care Fellows	
**PATIENT NAME:** Jane Doe<br>**PATIENT AGE:** 65<br>**CHIEF COMPLAINT:** Acute Dyspnea and Wheezing<br>**PHYSICAL SETTING:** Emergency Room	
Brief narrative description of the case	Jane Doe is a patient with a history of asthma presenting to the emergency department with acute respiratory failure, then rapidly deteriorates requiring initiation of mechanical ventilation. The case continues through different segments of her 12-day ICU stay with the patient developing dynamic hyperinflation, ARDS, and ventilator asynchrony prompting learners to recognize these and implement adjustments to the ventilator. In the last segment, the learner must assess the patient for readiness for ventilator weaning and liberation from the ventilator.
Primary learning objectives	By the end of this simulation case, fellows will learn about indications to intubate a patient with asthma exacerbation, use different ventilator modes and settings, immediate post-intubation care including ventilator complication prophylaxis or "ventilator bundles," interpreting the ventilator generated data (especially waveforms depicting dynamic hyperinflation and ventilator asynchrony), analyzing elevated peak vs plateau pressures, ventilator management in acute respiratory distress syndrome (ARDS), and ventilator weaning.
Critical actions	34-item critical action checklist listed in Supplementary Appendix B
Learner preparation	Prior to the initiation of the case, the learner is given the following information upon entering the room.<br>"Welcome to our mechanical ventilator simulation session. We will be taking you through some of the common case scenarios that you will see in the ICU. We will start by describing our set up: This is our patient, Jane doe, a high-fidelity manikin. This is the vital signs monitor. I will be the proctor taking you through the scenarios, operate the simulation system, will be speaking for the patient when needed, and assessing your performance. (ICU faculty) will also observe and assess your performance and act as the respiratory therapist when needed. Please interact with the manikin as you would with a real patient. You may ask questions and perform a physical exam on it. You may ask for medication/equipment/lab work/diagnostic imaging that you need, as you would do in real life. We ask that you share your thought process out loud to get credit for it. We understand that some things cannot be simulated. For this session to work, you need to suspend your notion of disbelief and assume you are with a real patient in real life. Do you have any questions?"
**Initial presentation**	
Initial vital signs	HR 135/min, BP 150/100 mm Hg, RR 25/min, afebrile and her SpO_2 91% (on non-rebreather mask 100% FiO_2)
Overall setting and appearance	The patient is represented by the high-fidelity Laerdal ALS manikin. Upon entering the simulation room, learners will notice the manikin sitting up at 90 degrees on a hospital bed, on a non-rebreather mask, with bedside monitor displaying vital signs. Two to four instructors are also present in the simulation room, each with different responsibilities. The lead instructor or proctor introduces the learner to the other instructor(s), their responsibilities, and equipment in the room, including a functioning Puritan Bennett 840 mechanical ventilators that can be used, if the learner decides to

(*Continued*)

 © 2023. The Author(s).

Supplementary Appendix A (*Continued*)

Actors and roles during a simulation session	**ICU fellow/primary learner:** The learner who will be responsible for directing the care of the patient during the session.<br>**The patient/manikin:** Represented by a manikin with voice portrayed by a simulation staff member (proctor) in the room.<br>**Clinical Educator track fellow:** Senior PCCM fellow will proctor the session, answer questions on behalf of the patient/manikin, operate and select various predesigned custom lung models on the ASL 5000 lung simulator and score the trainee's competency checklist;<br>**ICU faculty:** Board certified ICU faculty (among the co-authors of this project) who will observe the simulation session, act as the respiratory therapist, and rate learner on the competency checklist. This member also provides structured debriefing of the case interventions and feedback to the learner after the simulation session.<br>**Respiratory therapist (confederate):**<br>A faculty or simulation staff member should perform the role of the respiratory therapist when requested by the learner (connects or disconnects ETT to/from a ventilator, changes ventilator settings before the start of each segment (as listed in Supplementary Appendix B), implements changes to ventilator settings that learner requests. This role has certain standard scripted prompts as listed below.<br>**ICU nurse (confederate):**<br>The proctor or other available simulation staff member will play the role of ICU nurse when required. The role of the ICU nurse will be to give scripted updates on the patient should the segment/ learner requires.		
HPI	A 65-year-old female presented to the emergency department with acute dyspnea and wheezing. She has a history of severe persistent childhood asthma and had multiple admissions for acute exacerbations. She denies any phlegm, fever, chills, or chest pain. She reports having a sick toddler at home with runny nose and bronchitis. Since arrival, she has received IV steroids and continuous bronchodilators for 30 minutes in the emergency department. You are called to evaluate the patient in the emergency department for ICU admission.<br>**Additional information available if requested by the learner (provided by AA):**<br>Labs from the emergency department:<br>Complete blood count: within normal limits<br>Basic metabolic profile: within normal limits<br>Liver function tests: within normal limit<br>Coagulation studies: within normal limits<br>ABG: 7.28/65/62 (on the nonrebreather mask)		
Past medical/ surgical history	**Medications**	**Allergies**	**Family history**
Asthma	Patient unable to remember the names of her home inhalers	None	None
Physical examination			
General	Sitting up in bed, in severe respiratory distress, tachypneic, audible wheezing, unable to complete sentences		
Lungs	Bilateral diffuse wheezing and using accessory muscles		
Cardiovascular	Sinus tachycardia, S1 and S2 heard, no murmurs		
Abdomen	Soft, no organomegaly, normal bowel sounds		
Neurological	No focal neurological deficits		
Skin	Warm, no rash		

We created five unique modes on ASL 5000 for each of the six clinical scenarios that we wanted to test the learner:

1. Segment A: To depict normal lung with normal compliance and raw, with corresponding vital signs of BP 110/70 mm Hg, HR 96/min, RR 20/min, SpO_2- 96% on a ventilator.
2. Segment B: To depict dynamic hyperinflation, with normal compliance and raw, with corresponding vital signs of BP 90/45 mm Hg, HR 130/min, RR 39/min, SpO_2- 88% on a ventilator.
3. Segment C: To depict mucus plug causing elevated raw, with corresponding vital signs of BP 107/50 mm Hg, HR 140/min, RR 24/min, SpO_2- 89% on a ventilator.
4. Segment D: To depict ARDS with low lung compliance, with corresponding vital signs of BP 129/80 mm Hg, HR 117/min, RR 23/min, SpO_2- 85% on a ventilator.
5. Segment E: To depict double triggering lung-ventilator dyssynchrony, with normal compliance, with corresponding vital signs of BP 129/80 mm Hg, HR 98/min, RR 20/min, SpO_2- 96% on a ventilator.

 © 2023. The Author(s).

6. Segment F: To depict normal lung, with normal compliance and raw, with corresponding vital signs of BP 129/80 mm Hg, HR 98/min, RR 15/min, SpO_2- 96% on a ventilator.

The details for each of the six segments of the case, along with possible interventions/scripted lines for proctor/nurse/respiratory therapist are listed below.

Intervention/Time point	Change in case	Additional information
**Segment A:**<br>**(Initiating MV and immediate postintubation care)**<br>Beginning of scenario<br>Proctor selects segment A mode in ASL 5000 when learner chooses to initiate mechanical ventilation	After the learner completes history and brief physical exam on a patient, proctor asks: What would you like to do next?	● Based on learner's requests for Noninvasive vs invasive positive pressure ventilation, the proctor will select an appropriate mode on ASL 5000 and Respiratory therapist will connect manikin to ventilator settings per learner<br>● When the learner/scenario requires the patient to be intubated, ETT is placed in the manikin by the RT and connected to a ventilator.<br>● If at any point learner is unsure of the next steps: Proctor asks "Can you explain what you think is going on?." If the learner does not have the correct response, the case moves to the next critical action<br>● Height (if asked): 165 cm<br>● Weight (if asked): 70 kg
	Possible Interventions by the learner:<br>● CXR: Results are shown by the proctor (details in Supplementary Appendix C)<br>● ABG: 7.28/65/62<br>● Code status: Full code<br>● Noninvasive positive pressure ventilation (NIPPV): identify the indication and choice of NIPPV settings. Patients should be closely monitored.<br>● NIPPV initiated: Case move forward 30 minute and proctor asks "What would you like to do next?." Learners should do a physical exam (poorly responsive mental status) and request new ABG (shows worsening hypercapnic acidosis 7.22/72/58). The learner should choose to intubate patient due to clinical worsening<br>● Invasive positive pressure ventilation: identify indication. Proctor report that the emergency department team will intubate the patient electively.<br>● Initiate mechanical ventilation: RT asks the learner to list ventilator settings; proctor asks learner why a specific Vt was chosen<br>● Postintubation: Nurse asks "Are there any other orders you want now that patient is intubated"	
	12 Critical actions:<br>❖ Code status addressed before intubation<br>❖ Initiate mechanical ventilation<br>o If noninvasive ventilation is chosen, the learner should (a) monitor the patient closely clinically, (b) request repeat ABG, and (c) intubate patient at patient reassessment<br>o Identify indication to intubate: hypercapnic acidosis and respiratory distress in asthma exacerbation<br>❖ Postintubation care: Learner must initiate Vt at 6–8 cc/kg IBW, postintubation vital signs, ABG and CXR ordered and followed up on, sedation and analgesia addressed, ventilator prophylaxis bundle (elevate the head of bed to 30 degrees, chlorhexidine mouth wash, GI and VTE prophylaxis started).	
**Intervention/Time point**	**Change in case**	**Additional information**
**Segment B:**<br>**(auto-PEEP)**<br>Proctor selects segment B mode in ASL 5000<br>RT changes ventilator settings to 24/500/100/5, flow 60 LPM	The patient has now been moved to the ICU and you are called to assess the patient 90 min postintubation to assess ventilator alarms. Patient came with these ventilator settings (24/500/100/5) from the emergency department<br>Vitals:<br>RR 39/min, HR 130/min, BP 90/45 mm Hg, SpO_2- 88% on ventilator<br>Exam:<br>Awake, but agitated and restless, tachypneic.<br>Lung exam: bilateral diffuse wheezing and using accessory muscles. Poor air movement bilaterally	If at any point learner is unsure of the next steps: Proctor asks "Can you explain what you think is going on?." If the learner does not have the correct response, the case moves to the next critical action<br>• Height (if asked): 165 cm<br>• Weight (if asked): 70 kg

(*Continued*)

 © 2023. The Author(s).

(*Continued*)

	Possible interventions by the learner:<br>● ABG or CXR requested: results are pending<br>● Ventilator disconnected from ETT: performed by the learner or respiratory therapist on learner's request, will temporarily (for one min) improve BP to 100/60 mm Hg, HR to 120/min, and SpO_2 to 90% on ventilator settings.<br>● Ventilator changes:<br>o Both RR reduction to <10 and reduction of Vt by 2 cc/kg IBW need to be performed for auto-PEEP to resolve in this custom lung model.<br>o Increasing FiO_2 to 100% and/or PEEP to 10 will improve SpO_2 only marginally to 92%.<br>o Any other interventions: will not change vital signs<br>● Any of the 2 necessary MV interventions are not performed (reduce RR and Vt as per above): vital signs will worsen to HR 30/min, BP 60/40 mm Hg and SpO_2-80% over 2 min; and at this point, the nurse asks "What would you like to do with these worsening vital signs?." If the learner still does not implement the necessary MV changes, the nurse says "okay we can call the ICU attending" and the case moves to segment C.<br>● Sedation/Neuromuscular blockade: can be added if requested by the learner, but these alone will not resolve auto-PEEP during the time course of this module<br>● Bronchoscope requested: the proctor will report that a bronchoscope has been requested and is 15 min away	
	2 Critical actions:<br>❖ Diagnose unstable auto-PEEP (ventilator waveforms and clinical condition) and disconnect the circuit.<br>❖ Manage auto-PEEP by<br>o Making appropriate changes to MV settings (Low RR, low Vt)<br>o Use bronchodilator, consider sedation/neuromuscular blockade.	
**Segment C:**<br>**(mucus plug)**<br>Proctor selects segment C mode in ASL 5000<br>RT changes ventilator settings to 24/500/100/5, flow 60 LPM	Let us move to the next segment now. This is day 2 of the patient's ICU stay, the patient continues to be intubated, and you are called to assess the patient for ventilator alarms.<br>Vital signs:<br>HR 140/min, BP 107/50 mm Hg, SpO_2 89% on vent, RR 30/min<br>Exam:<br>Agitated, tachypneic.<br>Lung exam: Absent breath sounds on the right lung, wheezing on the left lung	If at any point learner is unsure of the next steps: Proctor asks "Can you explain what you think is going on?." If the learner does not have the correct response, the case moves to the next critical action
	Possible interventions by the learner:<br>● ABG requested: 7.38, 38, 56<br>● CXR requested: CXR shown to the learner (Supplementary Appendix C)<br>● Inspiratory pause check: normal plateau pressure, elevated peak pressure, and elevated R_{aw}<br>● Ventilator changes:<br>o Any increase in FiO_2 or PEEP will only marginally improve SpO_2 to 90–91%.<br>o Any other changes to ventilator settings (such as mode, RR, etc.) will not affect the vital signs or hypoxia.<br>● Ventilator tubing circuit checked: No kinks<br>● Patient's oral cavity checked: No biting of tube<br>● Respiratory cultures, bronchodilators, and antibiotics: Proctor says these will be performed/administered. No change in vital signs.<br>● Sedation: Proctor says these will be administered. No change in vital signs.<br>● Bronchoscope requested: the proctor will report that a bronchoscope has been requested and is 15 min away.	

 © 2023. The Author(s).

(*Continued*)

	● The learner is unsure of the next steps: Proctor asks "Can you explain what you think is going on?." If learner still does not diagnose mucus plugging, the nurse says "okay we can call the ICU attending" and the case moves to segment D<br>● Only some of the 4 interventions are mentioned: Proctor asks "Is there anything else we can do?." The segment ends now and the learner is scored based on interventions chosen by the learner. All 4 interventions should be mentioned.	
	2 Critical actions:<br>❒ Elevated airway pressure recognition by checking inspiratory pause<br>❒ Elevated airway pressure intervention–differential diagnosis and treatment plan<br>o Suctioning, chest physiotherapy, patient positioning, and bronchodilators	
**Segment D:**<br>**(ARDS)**<br>Proctor selects segment D mode in ASL 5000<br>RT changes ventilator settings to 12/500/60/5, flow 60 LPM	Let us move to the next segment now. This is now day 4 of the patient's ICU stay, the patient continues to be intubated and you are called to assess the patient to assess ventilator alarms and resolve them. Yesterday patient was diagnosed to have right-sided pneumonia with pseudomonas growing in respiratory cultures and the patient was started on appropriate antibiotics.<br>Vitals:<br>RR 23/min, HR 117/min, BP 129/80 mm Hg, SpO_2 85% on vent<br>Exam:<br>The patient is tachypneic, in moderate distress. Lung exam: bilateral diffuse crackles and using accessory muscles. No wheezing	If any of the 4 necessary interventions are not performed: hypoxia will persist<br>The learner should reduce Vt to 4–6 cc/Kg IBW (can reduce in increments of 1–2 cc/kg IBW) for lung-protective strategy<br>RR increased to target minute ventilation, with goal pH >7.15 on ABG, allow permissive hypercapnia
	Possible Interventions by the learner:<br>● ABG requested: 7.31, 56, 58<br>● CXR requested: results are shown to the learner (Supplementary Appendix C)<br>● Inspiratory pause check: elevated plateau pressure, normal R_{aw}<br>● ARDS diagnosed (based on ventilator waveforms and clinical scenario): Proctor asks "What would you do next?"<br>● Ventilator changes:<br>o Reduce Vt: Proctor asks "how much do you want to reduce Vt? And why?"<br>o RR increased: Proctor asks "how much do you want to increase RR? why?"<br>o increase in FiO_2 (even 100%) or PEEP will only marginally improve SpO_2 to 88–90%.<br>o Changing to PCV will not affect the vitals or hypoxia unless FiO_2 and PEEP are increased per ARDSNet table.<br>● The learner is unsure of the next steps: Proctor asks "Is there anything else you want to do?" If learner still does not mention all 4 interventions listed, the case moves to segment E<br>● Sedation: can be added if requested by the learner, but these alone will not resolve hypoxia during the time course of this module<br>● Bronchoscope requested: the proctor will report that a bronchoscope has been requested and is 15 min away.	
	1 Critical action:<br>❒ Initiate lung protective ventilation in ARDS<br>o Reduce Vt<br>o increase RR (<35) to maintain minute ventilation<br>o increase FiO_2 and PEEP per ARDSNet table and<br>o monitor plateau pressure (goal ≤ 30 cm H_2O)	

(*Continued*)

 © 2023. The Author(s).

(*Continued*)

**Segment E:**<br>**(ventilator dyssynchrony)**<br>Proctor selects segment E mode in ASL 5000<br>RT changes ventilator settings to 18/320/60/5, flow 70 LPM	Let us move to the next segment now. This is now day 8 of the patient's ICU stay, the patient continues to be intubated and you are called to assess the patient to assess ventilator alarms and resolve them.<br>Vitals:<br>RR 20/min, HR 98/min, BP 129/80 mm Hg, SpO_2 96% on ventilator<br>Exam:<br>Tachypneic, in moderate distress. No crackles or wheezing<br>Lung exam: bilateral normal sounds.	If any of the 4 necessary interventions are not performed: the dyssynchrony will persist on ventilator waveform
	Possible interventions by the learner:<br>● ABG or CXR requested: results are pending<br>● Learner unsure of the next steps: Proctor asks "What do you think is going on?." Learners should correctly identify the type of dyssynchrony (double triggering). Irrespective of the learner's answer, the proctor then moves to the next competency item and asks "Can you tell me how to resolve these alarms?"<br>● Ventilator changes:<br>o Increase Vt by $\geq$ 2 cc/kg IBW, reduce flow $\leq$40 LPM, consider changing mode to PCV, check/change trigger. If all of the changes are not performed, the dyssynchrony will persist and the proctor asks "Is there anything else you want to do?"<br>o Any other changes to ventilator settings including mode, RR, etc will not affect the vitals or hypoxia.<br>● Sedation: can be added if requested by the learner, but these alone will not resolve hypoxia during the time course of this module<br>● The learner is unsure of the next steps: Proctor asks "Is there anything else you want to do?" After the learner has attempted interventions he/she wants to, the segment concludes and the case moves to segment F	
	2 Critical actions:<br>❒ Identify the type of dyssynchrony (double triggering)<br>❒ Manage Dyssynchrony:<br>o increase Vt by $\geq$ 2 cc/kg IBW<br>o reduce flow $\leq$ 40 LPM,<br>o consider changing mode to PCV<br>o check/change trigger	
**Segment F:**<br>**(ventilator weaning)**<br>Proctor selects segment F mode in ASL 5000<br>RT: sets vent settings of 16/500/70/5, flow 60 LPM	Let us move to the final segment now. This is day 12 of the patient's ICU stay, the patient continues to be intubated and you are called to assess the patient for extubation.<br>Vitals:<br>RR 15/min, HR 98/min, BP 129/80 mm Hg, SpO_2 96% on vent<br>Exam:<br>Alert, follows commands, coughs and can lift her head of the bed.<br>lung exam: bilateral normal sounds.<br>NG tube feeding is very well tolerated.	Postextubation, RT removes ETT from the patient and turns off the ventilator.<br>Cuff leak should be considered for this patient given 12-day intubated stay, pneumonia, ARDS, 7.5 sizes ETT all increasing risk for tracheal inflammation. This manikin will demonstrate an adequate cuff leak.
	Possible interventions by the learner:<br>● ABG or CXR requested: results are shown by the proctor (Supplementary Appendix C)<br>● Hemodynamic status: Off vasopressors for >2 days now<br>● Ventilator settings: Learner should reduce FiO_2 to at least 50% and reassess the patient's oxygenation status by either SpO_2 or PaO_2 on ABG.<br>● Secretions/suctioning requirements: minimal secretions/no suctioning for last 6 hours<br>● Awakening trial/sedation: Patient has been off all sedatives for at least 4 hours	

 © 2023. The Author(s).

(*Continued*)

	● Neurological status: Patient is following simple commands<br>● Cough reflex: Present<br>● Tube feeding: The patient is currently running tube feeds. Learner should either hold tube feeds for 2–4 hours or can suction out gastric contents, before SBT.<br>● Raise the head of the bed: Performed by nurse/RT, if requested<br>● Learner unsure of the next steps: What is the next thing you would want to do?<br>● SBT requested: Proctor asks "what do you mean by SBT? Can you explain to me/ demonstrate how to place the patient in SBT?." Learners should correctly choose the mode (spontaneous) and place the patient on PS 5–8 cm H_2O and PEEP 5 cm H_2O with $FiO_2 \leq 50\%$. Irrespective of the learner's correct/ incorrect performance, proctor next asks learner how to interpret SBT<br>● SBT initiated: "How long do we keep patients on SBT? What parameters are you assessing the patient on the ventilator?." The learner has to calculate RSBI correctly and note that RSBI <105 for this patient<br>● SBT interpretation completed: "What is the next step?"<br>● Cuff leak test: Learner asked to explain/demonstrate the cuff leak test. The learner has to demonstrate cuff leak by either the auscultatory method or Vt method (>105 cc difference). If using the Vt method, the learner has to perform a cuff leak in AC/VC mode.<br>● Extubate patient: RT says "Okay, I can extubate the patient. Is there anything else you want me to do after the patient is extubated?." While nasal cannula (to titrate to SpO_2) is adequate for this patient, other acceptable responses include NIPPV or high flow (provided learner explains rationale like hypercapnia on admission or significant hypoxia, etc.), followed by monitoring to wean down to nasal cannula.<br>● Post extubation: Proctor says "It has been 6 hours since extubation and the nurse wants to know if we can transfer the patient out of the ICU to the medical floor because we need an ICU bed for another patient. The patient has been doing well for the last 6 hours post-extubation."<br>● Transfer patient out of ICU: Case ends<br>● No, do not transfer the patient out of ICU: Proctor asks "Why not? And when can we transfer the patient?." The learner should identify monitoring for stridor and respiratory distress/ reintubation as reasons to monitor the patient for at least 24 hours postextubation (given patient's prolonged ICU course)	
	15 Critical actions:<br>❒ Evaluate patient for SBT<br>o Primary indication to intubate patient has resolved (asthma/pneumonia/ARDS)—determined by CXR, ABG, normal lung auscultation<br>o Hemodynamic stability<br>o Recent ABG reviewed<br>o Assess ventilator settings<br>o Assess for secretions/suctioning requirements<br>o Awakening trial<br>o Neurological status<br>o Discontinue tube feeds<br>o Raise the head of the bed<br>❒ Perform SBT<br>❒ Interpret SBT<br>❒ Prepare for extubation	

(*Continued*)

 © 2023. The Author(s).

(*Continued*)

	o Perform cuff leak<br>o Interpret cuff leak tests<br>❒ Post extubation<br>o Oxygen supplementation<br>o Monitor for 24 hours postextubation for complications

Abbreviations: ABG, arterial blood gas (listed as pH, $PaCO_2$, PaO_2); AC/VC, assist control/volume control; ARDS, acute respiratory distress syndrome; CXR, chest X-ray; ETT, endotracheal tube; FiO_2, fraction % of inspired oxygen; GI, gastrointestinal; HR, heart rate; IBW, ideal body weight; ICU, intensive care unit; LPM, liters per minute; MV, mechanical ventilation; NIV, noninvasive positive pressure ventilator; PaO_2, partial pressure of arterial oxygen content; PCCM, pulmonary and critical care medicine; PCV, pressure control mode; PEEP, positive end expiratory pressure; PS, pressure support; RR, respiratory rate; RSBI, Rapid Shallow Breathing Index; RT, respiratory therapist; SBT, spontaneous breathing trial; SpO_2, oxygen saturation via pulse oximetry; Vt, tidal volume; VTE, venous thromboembolism.
Note: Ventilator settings are listed as RR/Vt/ FiO_2/PEEP.

Ideal Scenario Flow

The learner enters the room to find a patient in respiratory distress. Simulation team member AA introduces the learner to the case, personnel, and equipment. Learner recognizes that patient is hypoxic on bedside monitors, on a non-rebreather mask. Based on clinical presentation, appropriate history, physical examination, and laboratories, the learner decides either between invasive or noninvasive mechanical ventilation for asthma exacerbation. After this, the learner is taken through six clinical scenarios of a critical ill asthma patient in the ICU (intubation, dynamic hyperinflation, ventilator asynchrony, mucus plug causing elevated airway resistance pressures, hypoxia with elevated plateau pressures suggestive of ARDS, and ventilator weaning), each in different time points of the patient's ICU hospital stay. The five clinical scenarios are listed as segments A–F in Supplementary Appendix B: Critical Action (Mechanical Ventilation Competency Checklist), each occurring at different periods of this hypothetical patient's hospital admission.

Anticipated Management Mistakes

Failure to follow ARDSnet protocol for ARDS management: Many of our learners didn't calculate the ideal body weight and instead used the body weight in kilograms to calculate the tidal volume and therefore failed to correctly use ARDSnet table for ARDS management. Some of the learners also didn't adjust the respiratory rate with low tidal volume ventilation for ARDS to match the same minute ventilation to avoid decompensation from acute hypercapnic respiratory failure.

Inability to check autopeep accurately: Some of the learners performed inspiratory pause to check for the autopeep instead of expiratory pause.

Failure to perform cuff leak test accurately: While performing cuff leak test for evaluating patient's readiness for extubation, many learners kept the patient on the spontaneous mode instead of the volume control mode. We created debriefing materials to cover the aforementioned management mistakes.

 © 2023. The Author(s).
