# Supplementary material for: A Pilot Standardized Simulation-Based Mechanical Ventilation Curriculum Targeting Pulmonary and Critical Care Medicine and Critical Care Medicine Fellows
Source: Avicenna J Med. 2023 Oct 3;13(3):176–81. doi: 10.1055/s-0043-1773792 (PMC10550363; doi:10.1055/s-0043-1773792)
Supplement: Supplementary file 2 — Supplementary Appendix B [file 10-1055-s-0043-1773792-s236b.pdf]

Supplementary Appendix B

Multiple Choice Questions- Mechanical Ventilation

Participant's name:
Year in training:
Date:

1. A 20-year-old man with ARDS due to viral pneumonia is requiring mechanical ventilator support. He was started on the following ventilator settings of: AC, VT 12 mL/kg, and a respiratory rate of 12. He is on 15 cm H_2O PEEP and a FiO_2 of 0.5. His plateau pressure of 40 cm H_2O and ABG are as following: pH is 7.39, Pco_2 is 40 mm Hg, and Po_2 is 89 mm Hg. You wish to follow the NIH ARDS Network protocol and, thus, at this point you should do all the following **EXCEPT**:
   a. Reduce the tidal volume to 6 mL/kg (PBW).
   b. Increase respiratory rate to 28.
   c. Increase PEEP to improve Po2
   d. Target Pplateau <30 mm Hg.

2. A patient with severe angioedema was intubated to secure airway and was started on the pressure control (PC) with the following settings: Pi 13, PEEP 5, Ti 1.3, and RR of 14. He is alert oriented and wrote to the nurse that he is not comfortable taking breaths.

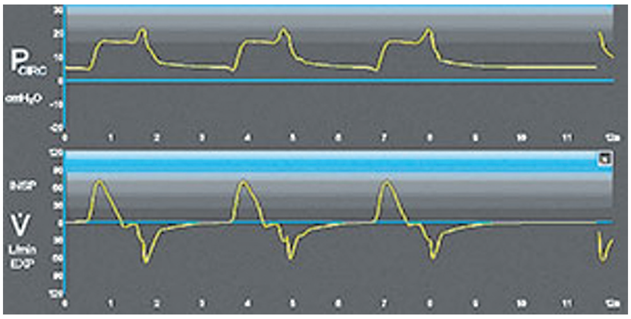

What is the first appropriate change you can do to improve his synchrony and comfort.
   a. Reduce inspiratory time (Ti).
   b. Increase sedation.
   c. Switch to volume control (VC) .
   d. Measure plateau pressure (Pplt).

3. A 65-year-old man required mechanical ventilation for a severe COPD exacerbation. After few days of ventilator support along with intense bronchodilator therapy, corticosteroids, and antibiotics, his respiratory status has improved and his FiO_2 requirements are now only 0.3 with 5 cm H_2O PEEP. A spontaneous breathing trial (SBT) is attempted, but he becomes slightly agitated, ABG was ordered and showed pH 7.32, PaCO2 55, and PaO2 of 85. His rapid shallow breathing index (RSBI) was 90, and the nurse held sedation and tube feeding 4 hours ago. Currently, he follows commands and has strong cough. What would you do next ?
   a. Extubate to noninvasive ventilation.
   b. Stay on ventilator until has lower RSBI <85.
   c. Extubate to high flow nasal cannula.
   d. Extubate if repeat BAG shows pH >7.35

4. A patient with ARDS has the chest CT scan (figure below) at end exhalation with 5 cm H_2O PEEP. Compared with the lower half of the lungs (dependent regions), the upper half of the lungs (non-dependent regions) during a positive pressure breath will:

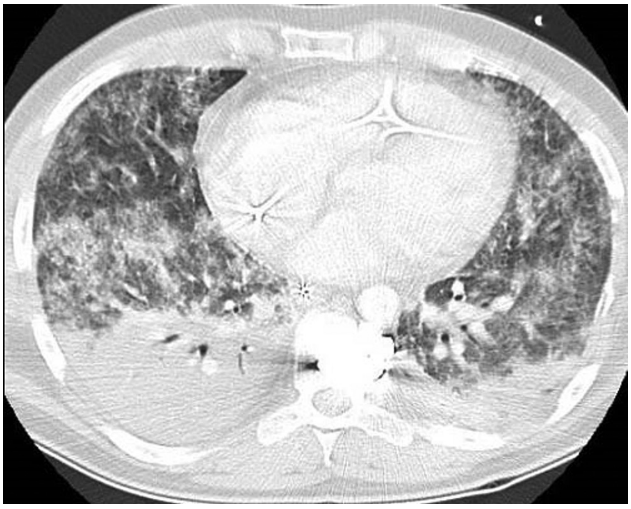

   a. Vulnerable to volutrauma
   b. Vulnerable to Barotrauma
   c. Less compliant
   d. Has more resistance.

5. Which ventilator strategies will result in increased PaCO2 compared with the control group?
   a. Airway pressure release ventilation (APRV)
   b. Prone positioning
   c. Inhaled nitric oxide
   d. Using neuromuscular blockers

6. During airway pressure control ventilation PCV with driving pressure of 25 cm H_2O and PEEP set at 5 cm H_2O. He is obese and his esophageal sensor registering pleural pressure of +7 cm H_2O during inspiration. What is the ultimate end inspiratory trans-pulmonary pressure?
   a. Similar to the driving pressure of 25 cm H2O

 © 2023. The Author(s).

b. Similar to the driving pressure of 25 cm H2O minus the patient generated intrapleural pressure of +-7 cm H2O = 36 cm H2O

c. Similar to the Plateau pressure of 30 cm H2O minus patient generated intrapleural pressure of +7 cm H2O = 23 cm H2O

d. Similar to the PEEP of 5 cm H2O.

7. What mode of MV is this?

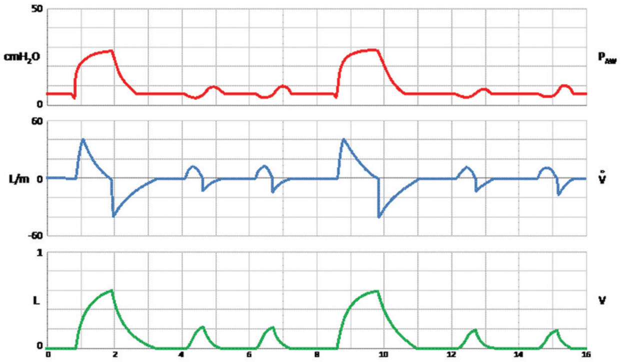

a.VCV b. PCV **c.SIMV** d. PSV

8. What mode of MV is this?

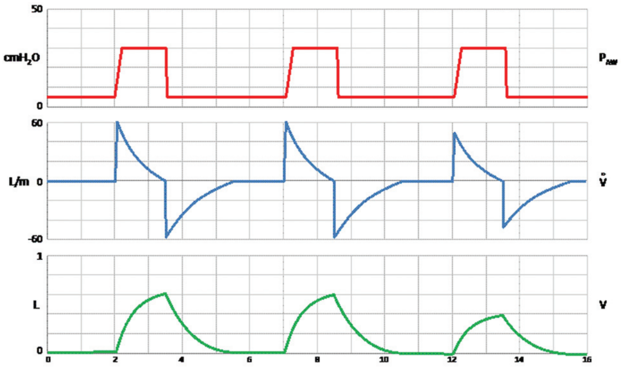

a.VCV **b.PCV** c.SIMV d.PSV

9. In a morbidly obese patient receiving mechanical ventilation, the end-inspiratory airway plateau pressure (Pplat) of 40 cm H_2O is likely to:

Select one:

a. Causes volutrauma
b. Be a gross underestimate of end-inspiratory transpulmonary pressure
c. Be a gross overestimate of end-inspiratory transpulmonary pressure
d. Causes barotrauma

 © 2023. The Author(s).

10. A patient status post-pneumonectomy 3 years ago is requiring mechanical ventilatory support in the ICU following complicated ruptured appendicitis and ARDS. You have a ventilator management protocol in your ICU that has target values for tidal volume of 6 mL/kg ideal body weight, rate titrated to pH > 7.30, and PEEP 5–15 cm H_2O. The measured plateau pressure was 37 mm Hg. What would you do to reduce Plateau pressure < 30?
    a. Use lower tidal (3 - 4 ml/kg).
    b. Use lower respiratory rate.
    c. Avoid high PEEP.
    d. Bronchodilators.

11. 70-year-old woman with cirrhosis and moderate ascites was intubated for acute hypoxic respiratory failure and community acquire pneumonia. Initial ventilator settings were; Vt 500, RR 14, FiO2 0.8 and PEEP 5 cm H2O. 12 hours after intubation, her ventilator alarm goes off indicating peak pressure of 50 and plateau pressure was 28. What is the likely etiology of high airway pressure?
    a. ARDS
    b. Increased secretions and mucus plugging
    c. Worsening ascites
    d. Pleural effusion

12. When elastance and resistance are changed in respiratory system and Volume and flow remain constant as pressure vary, what kind of control is this?
    a. Time control ventilation
    b. Pressure control ventilation
    c. Volume control ventilation
    d. B + A

13. When comparing pressure control (PCV) and PRVC (VC +), which statement is true:
    a. No difference
    b. VC+ guarantees tidal volume regardless of lung compliance
    c. Only PCV can control inspiratory time
    d. In VC + , ventilator determines driving pressure (Pi)

14. 67-year-old female (weight 50 kg) with severe emphysema is admitted for COPD decompensation. She failed NIPPV and required sedation, paralysis, and intubation. Soon after intubation and initiation of mechanical ventilation, she became hypotensive (BP dropped from 170/95 to 80/60). She has cold extremities, distended neck veins, midline trachea, distant heart sounds, and symmetrical breath sounds with prolonged expiratory phase.

Ventilatory settings are: assist control, tidal volume 500ml, rate 15/min, PEEP 5 cm H_2O, FIO_2 1.0 (100%) ABG: pH 7.20, PaO_2 250 mm Hg, $PaCO_2$ 77 mm Hg. Measured variables: rate 15/min, VE = 7.5 l/min, Ppeak 45 cm H_2O, Pplat 30 cm H_2O
The next step in this patient's management should be:

A. Order a stat echocardiogram to assess for tamponade.
B. Order a stat AngioCT to assess for pulmonary embolism.
C. Measure AutoPEEP, disconnect the patient briefly from the ventilator, then resume ventilation with a lower tidal volume and rate and administer intravenous fluid.
D. Start the patient on intravenous dopamine and adjust the ventilator to normalize the PaCO2.

15. NIV can be used in the following patients except:
    a. COPD exacerbation
    b. Pulmonary edema
    c. Pneumonia in immuno-compromised subjects
    d. Myasthenia gravis with significant bulbar weakness.

 © 2023. The Author(s).

Mechanical Ventilation Competency Checklist

Fellow's name:
Year in training:

Location: Date:

Evaluator name:

Starting time: End time:

Key: A= Completed B = Deficient

**Segment A (Initiating MV)**	**A**	**B**
Indication for mechanical ventilation identified (Invasive versus non-invasive)		
Address code status (Discuss with patient/family or review chart)		
**Segment A (Immediate post-intubation care)**<br>**MV settings: as requested by learner**		
Vt (IBW consideration)		
Check vital signs after intubation		
Order CXR after intubation to assess the ETT position		
Order ABG (after intubation)		
Assess need for sedation & analgesia<br>(immediately after intubation)		
Follow up on ABG		
Follow up on CXR		
Elevate HOB at least 30%		
Chlorhexidine mouth wash		
VTE/GI prophylaxis		
**Segment B (Auto-PEEP assessment): Day #1 (90 minute post intubation)**<br>**MV settings: RR 24, Vt 500,** FiO_2 **100% and PEEP 5**		
Unstable auto-PEEP recognition and disconnecting the circuit.		
Manage auto-PEEP by making appropriate changes to MV settings (Low RR, low Vt),<br>use bronchodilator, consider sedation		
**Segment C (Airway pressure assessment)**<br>**MV settings: RR 24, Vt 500,** FiO_2 **100% and PEEP 5**		
Elevated airway pressure recognition by checking inspiratory pause		
Airway pressure alarms intervention<br>(Provide differential diagnosis and treatment<br>plan-suctioning, chest physiotherapy, patient positioning and bronchodilators)		
**Segment D (Lung protective ventilation in ARDS)**<br>**MV settings: RR 12, Vt 500,** FiO_2 **60% and PEEP 5**		
Initiate Lung protective ventilation in ARDS<br>{Reduce Vt, increase RR to maintain minute<br>ventilation, increase PEEP using ARDSnet guidelines (1), monitor plateau pressure<br>(goal $\leq$ 30cm H_2O)}		
**Segment E (Patient-ventilator dys-synchrony)**<br>**MV settings: RR 18, VT 320,** FiO_2 **60%, PEEP 5 with flow 70LPM.**		
Dys-synchrony recognition (double triggering)		
Dys-synchrony intervention<br>(Change Vt, change flow, change mode, check/change trigger)		

 © 2023. The Author(s).

(*Continued*)

Segment A (Initiating MV)	A	B
**Segment F (Weaning MV)**<br>**MV settings: RR 16, VT 500, FiO_2 70% and PEEP 5**		
Assess readiness for weaning (Reexamining indication for MV)		
Assess hemodynamic stability (vital signs)		
Review recent ABG (or) order new ABG		
Assess FiO_2 & PEEP requirement<br>(O2 Saturation $>$ 90%, and PaO2 $>$60)		
Assess for secretions/ suctioning requirement		
Assess awakening trial (sedation interruption)		
Assess neurological status and alertness (following commands and cough reflex)		
Discontinue tube feeding before extubation		
Raise the head of bed prior to extubation		
Consider SBT and assess for the duration of the trial (at least 30 minute trial)		
Interpreting RSBI (uses correct ratio of RR/Tv and cut off value of 105)		
Consider a cuff leak test before extubation		
Provided correct cuff leak technique (listening for an air leak or recognizing adequate air leak on MV on assist control mode of ventilation)		
Considering Oxygen supplementation/NIV/ HFNC if needed post-extubation		
Assess patient for stridor and/or distress for 24 hours post-extubation		

Abbreviations: ABG, Arterial blood gas; ARDS, acute respiratory distress syndrome; ARDS, Acute respiratory distress syndrome; CXR, Chest X-ray; ETT, endotracheal tube; FiO_2, Fraction of inspired oxygen; GI, Gastrointestinal tract; HFNC, High flow nasal cannula; IBW, ideal predicted body weight; MV, Mechanical ventilation; NIV, Non-invasive ventilation; O2, Oxygen; PaO2, partial pressure of alveolar oxygen; PEEP, Positive end-expiratory pressure; RR, Respiratory rate; RSBI, Rapid shallow breathing index; SBT, Spontaneous breathing trial; Vt, Tidal volume; VTE, Venous thromboembolic events.

Satisfaction Assessment

Participant's name:
Year in training:
Date:

Rate the following on a 1–5 Likert scale (1-very poor, 2-poor, 3-acceptable, 4-good, 5-excellent)
The course helped your confidence and understanding of:

- Indications for Mechanical ventilation ________
- Initial settings of mechanical ventilation ________
- The basic modes of mechanical ventilation ________
- Managing airway's pressure alarms ________
- AutoPEEP and airflow limitation strategy ________
- Concepts of Lung Protective Ventilation (LPV) ________
- Patient ventilator dyssynchrony ________
- Weaning / Liberation protocol ________

Thoughts on the simulation training environment:

- Time was adequate ________
- The scenario represented a real-life situation ________
- Simulation laboratory was safe ________
- Debriefing was constructive] ________
- Timing and scheduling were accommodating ________
- I would like to participate in another simulation experience ________

 © 2023. The Author(s).
