# Supplementary material for: A Pilot Standardized Simulation-Based Mechanical Ventilation Curriculum Targeting Pulmonary and Critical Care Medicine and Critical Care Medicine Fellows
Source: Avicenna J Med. 2023 Oct 3;13(3):176–81. doi: 10.1055/s-0043-1773792 (PMC10550363; doi:10.1055/s-0043-1773792)
Supplement: Supplementary file 3 — Supplementary Appendix C [file 10-1055-s-0043-1773792-s236c.pdf]

Appendix C

Mechanical Ventilator Simulation Instructor Module And Debrief

A

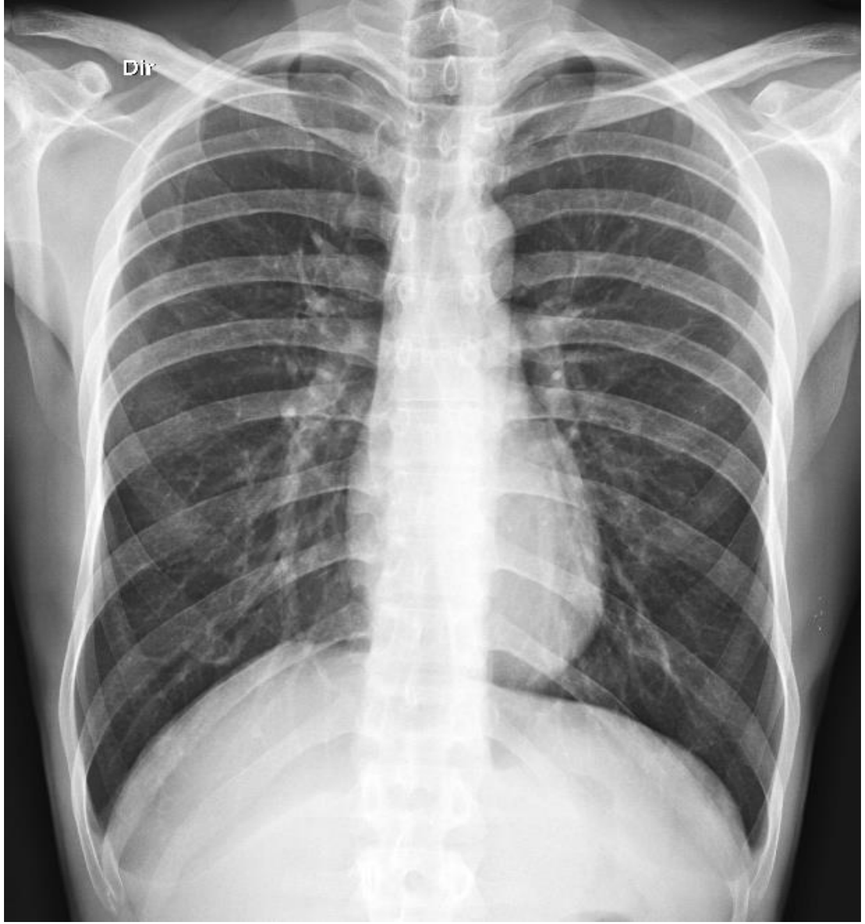

https://radiopaedia.org/licence?lang=us

Case courtesy of Dr Bruno Di Muzio, Radiopaedia.org, rID: 37906 with use under Creative Commons License

A

Patient height is 165 cm

Refer to ARDSnet table for predicted body weight per gender

http://www.ardsnet.org/tools.shtml

A

Post intubation CXR

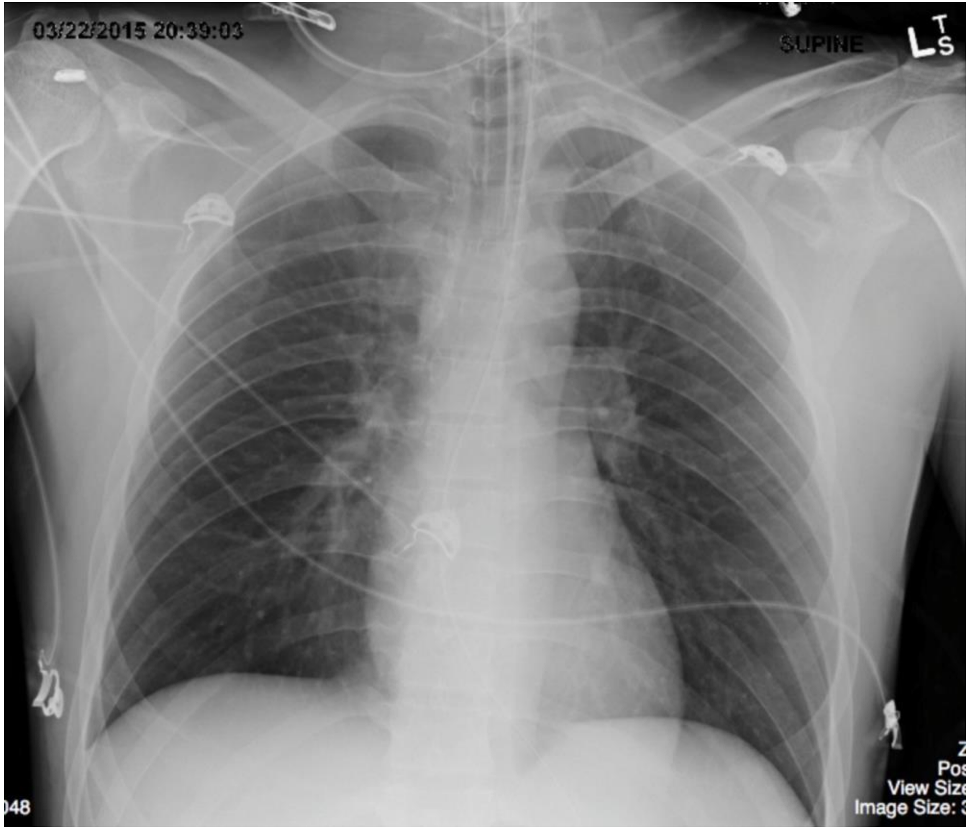

https://undergradimaging.pressbooks.com/chapter/tubes-and-catheters/

Curtesy of Brent Burbridge, with use under Creative Commons License

90min after intubation

B

B

Objective	
**Vital signs**	HR 130/min , BP 90/45 mmHg, RR 39/min , SpO2- 88% on ventilator
**Patient exam**	• Awake, Agitated and restless, tachypneic.<br>• Lung exam: Bilateral diffuse wheezing & using accessory muscles. Poor air movement bilaterally
**Vent alarms**	Elevated peak and plateau pressures
**Diagnostics**	ABG and CXR are pending (if learner asks)

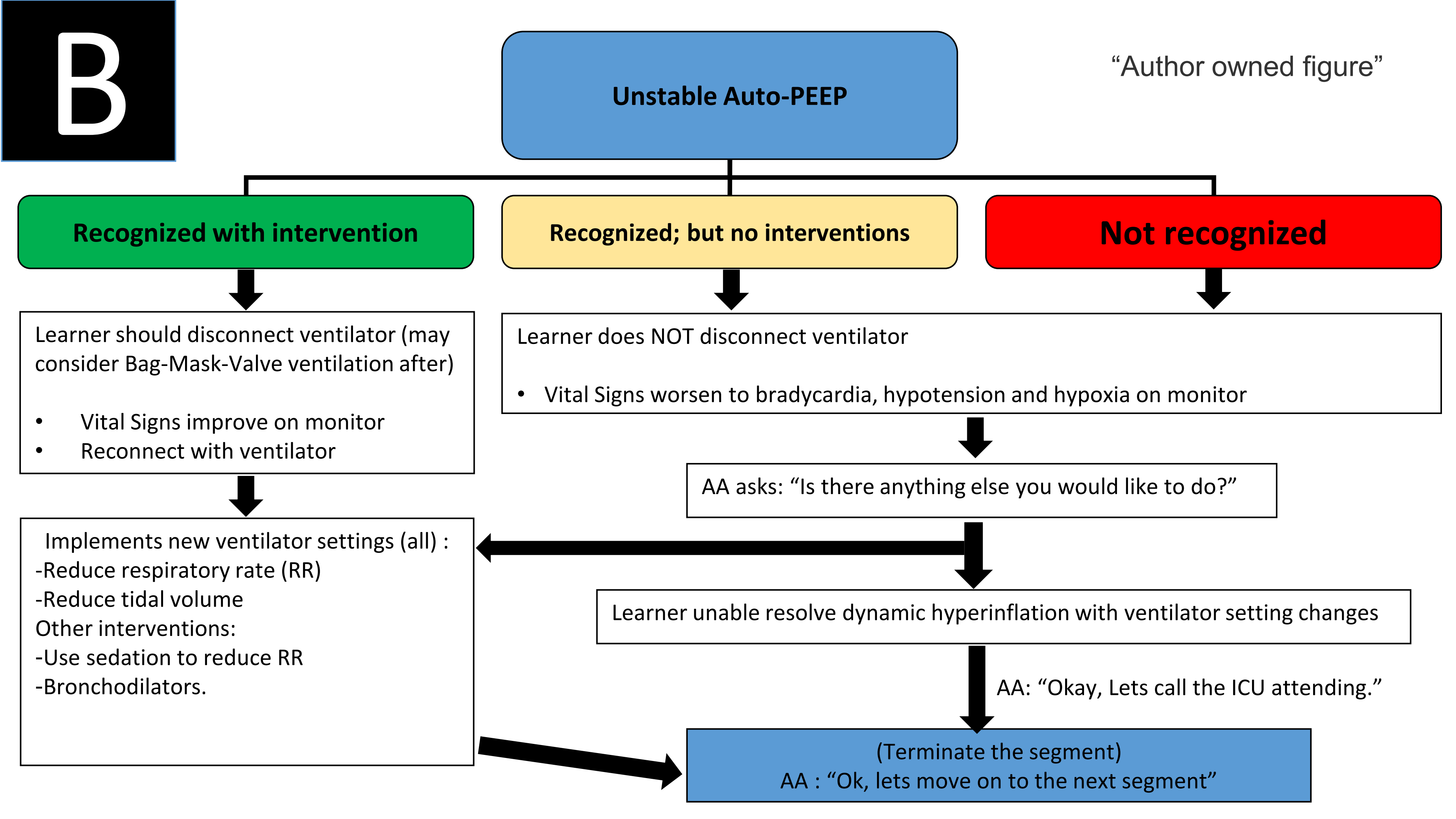
B
"Author owned figure"
Unstable Auto-PEEP
Recognized with intervention
Recognized; but no interventions
Not recognized
Learner should disconnect ventilator (may consider Bag-Mask-Valve ventilation after)
• Vital Signs improve on monitor
• Reconnect with ventilator
Learner does NOT disconnect ventilator
• Vital Signs worsen to bradycardia, hypotension and hypoxia on monitor
AA asks: "Is there anything else you would like to do?"
Implements new ventilator settings (all) :
-Reduce respiratory rate (RR)
-Reduce tidal volume
Other interventions:
-Use sedation to reduce RR
-Bronchodilators.
Learner unable resolve dynamic hyperinflation with ventilator setting changes
AA: "Okay, Lets call the ICU attending."
(Terminate the segment)
AA : "Ok, lets move on to the next segment"

Day #2 admission

C

C

Objective	
Vital signs	HR 140/min, BP 107/50 mmHg, RR 30/min**,** SpO2- 89 % on ventilator
Patient exam	Agitated, tachypneic<br><br>Lung exam: Absent breath sounds on right lung, Wheezing on left lung
Vent alarms	High peak pressure, low plateau , loud alarms.
Diagnostics	ABG (if asked by learner) from earlier today: 7.38, 38, 56<br>CXR (if asked by learner) on next page

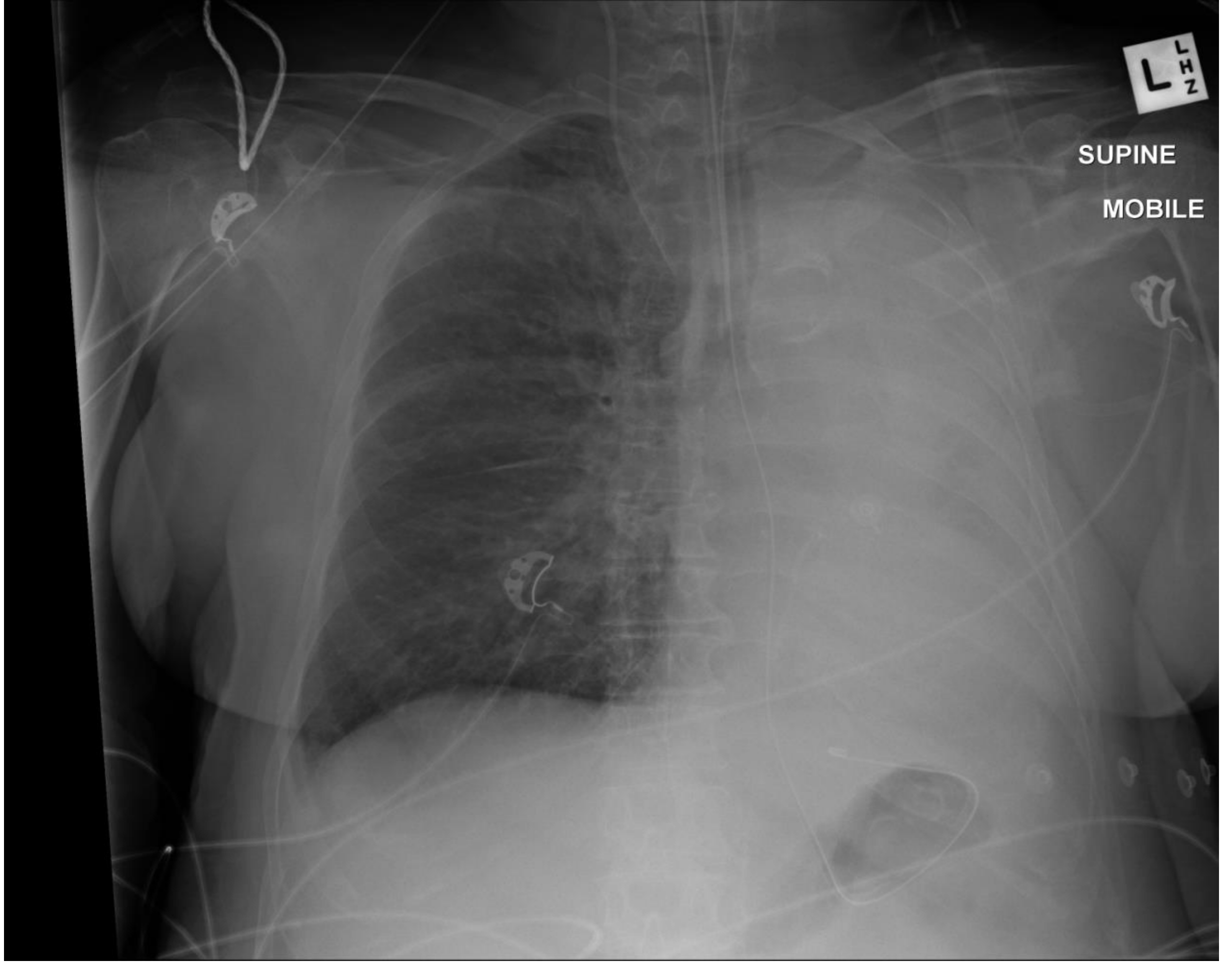

C

https://radiopaedia.org/licence?lang=us

Case courtesy of Dr Brendon Friesen, Radiopaedia.org, rID: 38171 under Creative Commons license

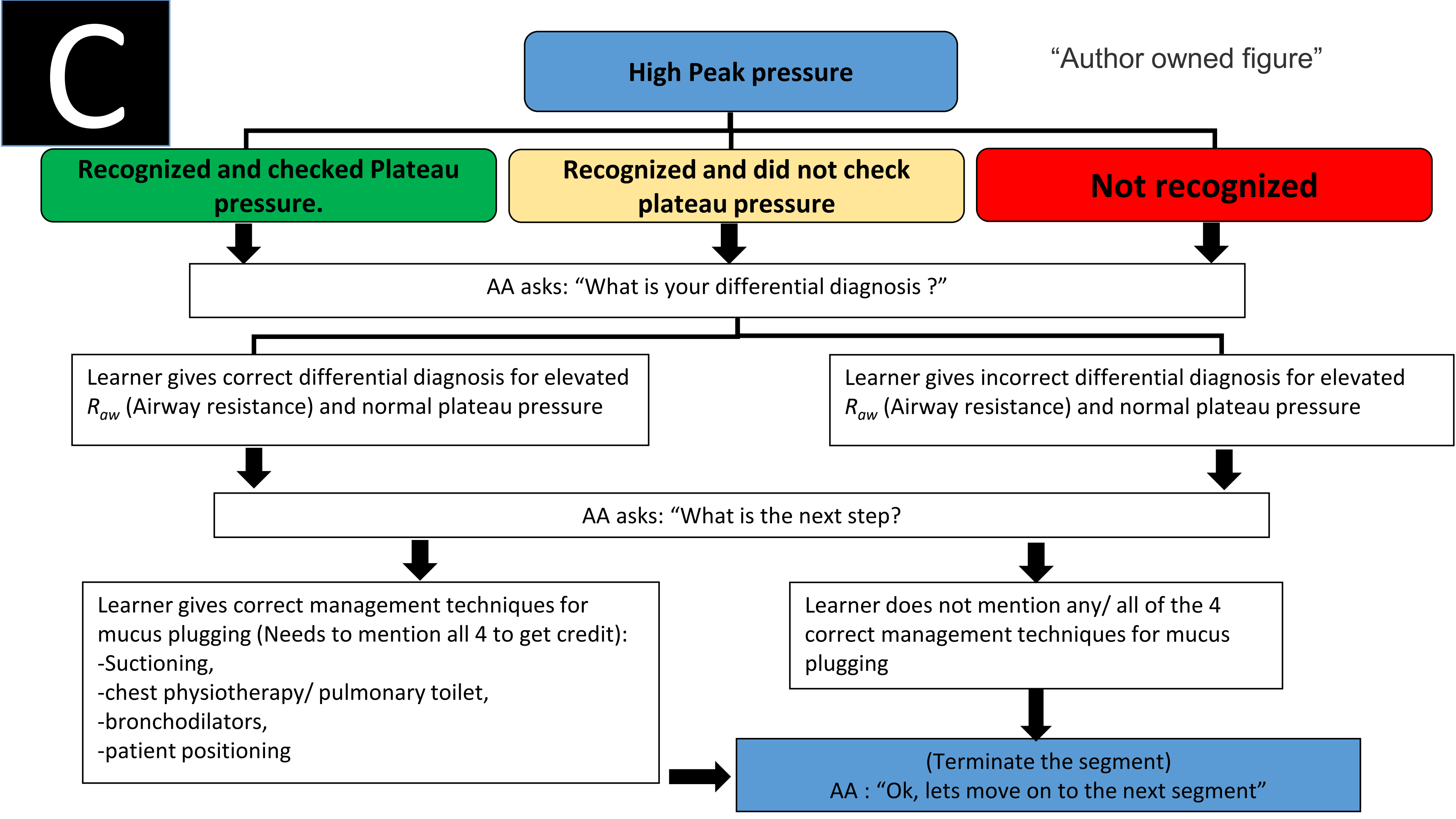
C
"Author owned figure"
High Peak pressure
Recognized and checked Plateau pressure.
Recognized and did not check plateau pressure
Not recognized
AA asks: "What is your differential diagnosis ?"
Learner gives correct differential diagnosis for elevated R_{aw} (Airway resistance) and normal plateau pressure
Learner gives incorrect differential diagnosis for elevated R_{aw} (Airway resistance) and normal plateau pressure
AA asks: "What is the next step?
Learner gives correct management techniques for mucus plugging (Needs to mention all 4 to get credit):
-Suctioning,
-chest physiotherapy/ pulmonary toilet,
-bronchodilators,
-patient positioning
Learner does not mention any/ all of the 4 correct management techniques for mucus plugging
(Terminate the segment)
AA : "Ok, lets move on to the next segment"

Components of Inflation Pressure

Image courtesy of Mazen Kherellah, MD, FCCP
Permission for use under Attribution-NonCommercial CC license

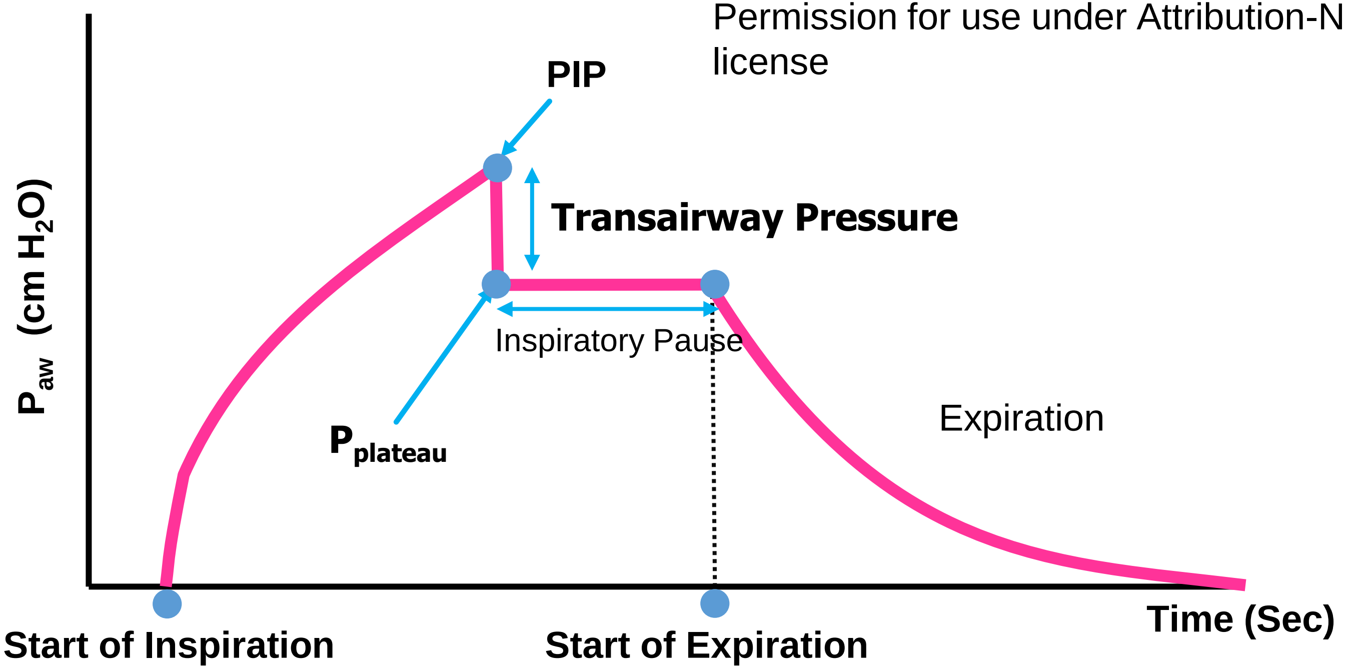

Respiratory Dynamics

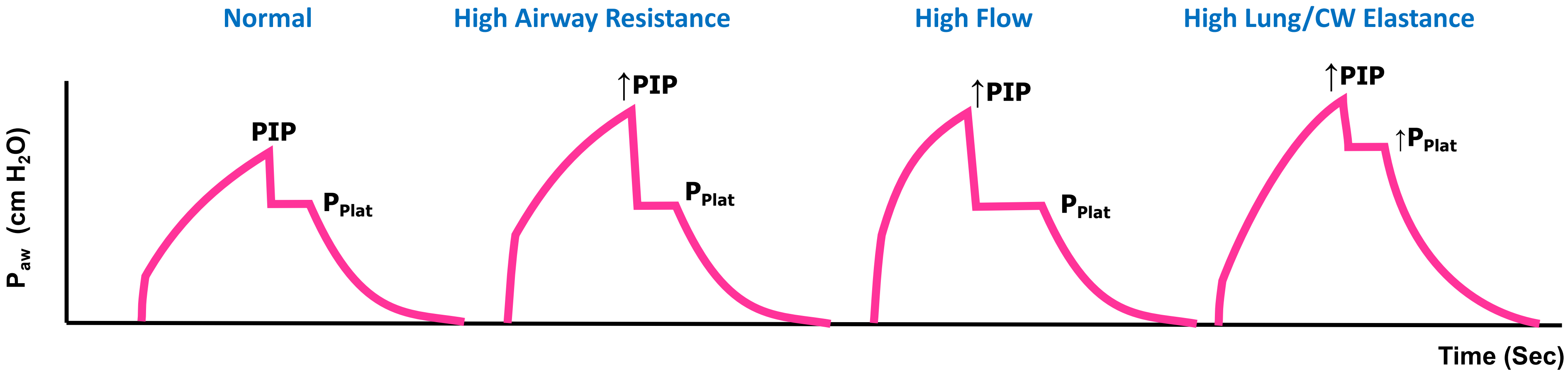

Image courtesy of Mazen Kherellah, MD, FCCP
Permission for use under Attribution-NonCommercial CC license

Day #4 admission

D

D

Objective	
**Vital signs**	HR 117/min, BP 129/80 mmHg, RR 23/min, SpO2 -85% on ventilator
**Patient exam**	Patient is tachypneic, in moderate distress.<br>Lung exam: Bilateral diffuse crackles & using accessory muscles. No wheezing
**Vent alarms**	High peak and plateau pressures
**Diagnostics**	ABG : 7.31, 56 , 58 (if asked by learner)<br>CXR (if asked by learner) on next page

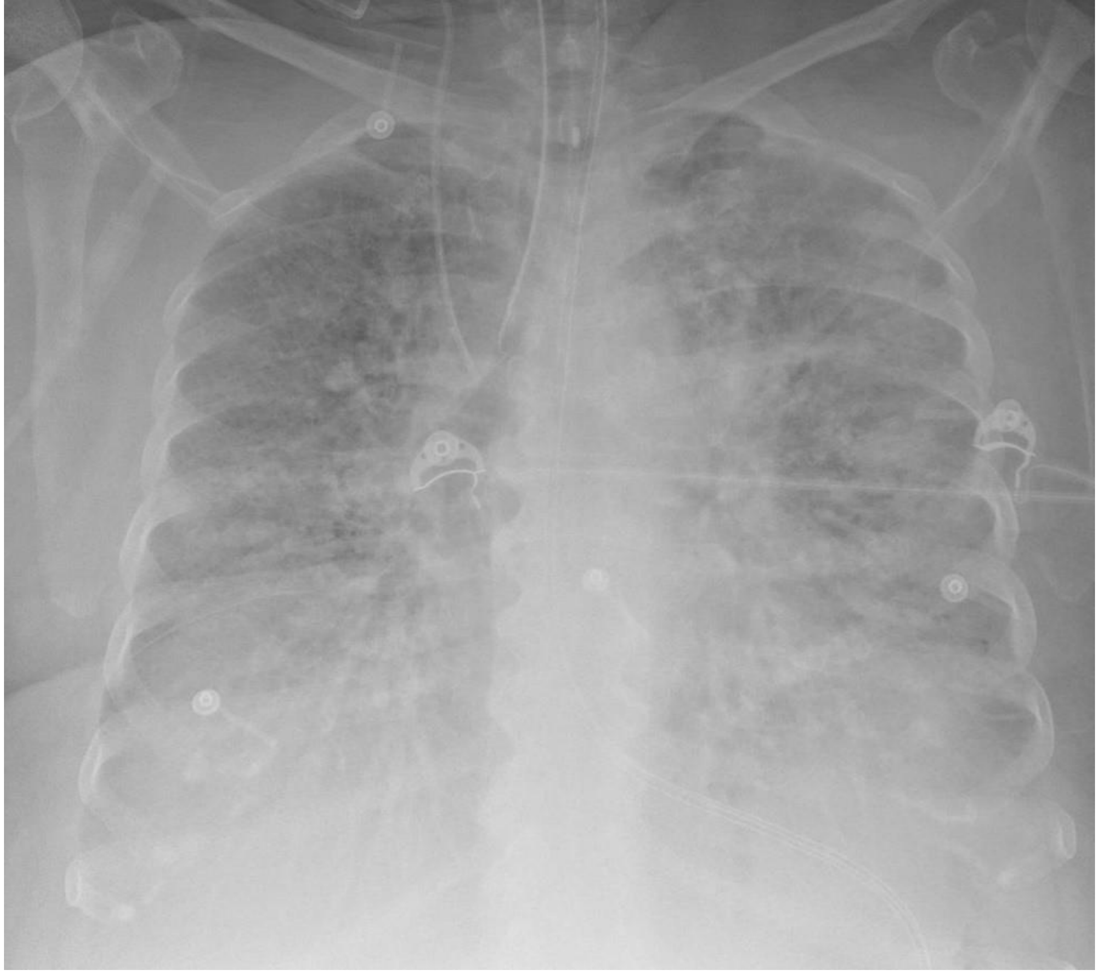

D

https://radiopaedia.org/licence?lang=us

Case courtesy of Dr Edgar Lorente, Radiopaedia.org, rID: 75182 use under Creative Commons License

D

Refer to ARDSnet table for oxygenation goals and PEEP tables

http://www.ardsnet.org/tools.shtml

D

Refer to ARDSnet table for pH goal, I:E goal and spontaneous breathing trial

http://www.ardsnet.org/tools.shtml

Refer to ARDSnet table for predicted body weight (PBW) per gender

http://www.ardsnet.org/tools.shtml

D

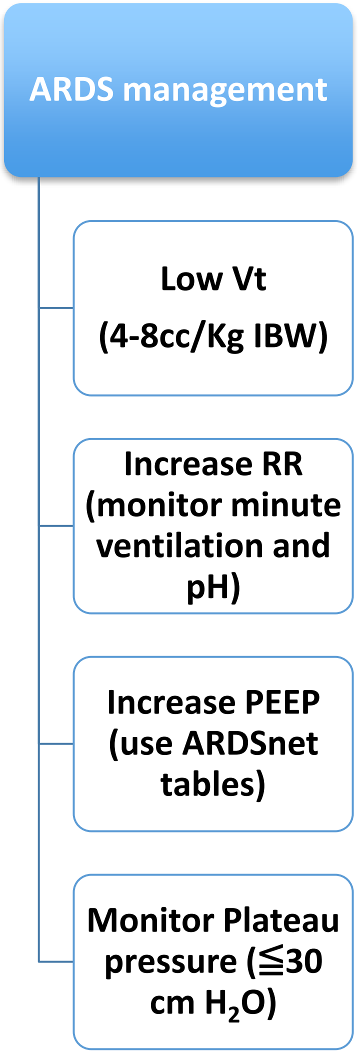

"Author owned figure"

Day#8 of admission

E

E

Objective	
**Vital signs**	HR 98/min, BP 129/80 mmHg , RR 20/min , SpO2- 96% on ventilator
**Patient exam**	Tachypneic , in moderate distress. No crackles or wheezing<br>Lung exam: Bilateral normal sounds.
**Vent alarms**	Cycle dys-synchrony (double triggering/ double stacking)
**Diagnostics**	ABG (if learner asks) : 7.38/51/98 on ventilator settings of 16/350/60/8<br>CXR (if learner asks): pending

Types of Patient-Ventilator Dys-synchrony

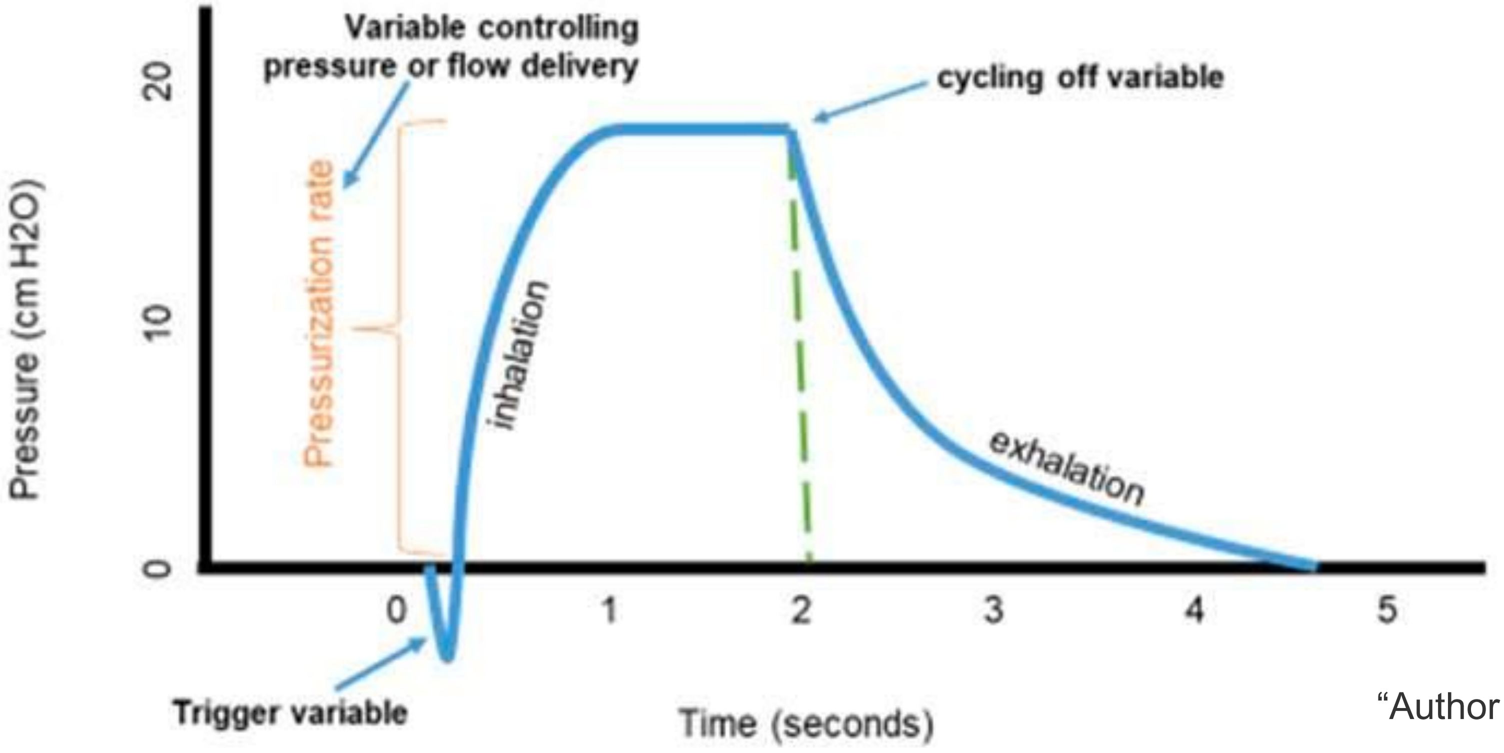

"Author owned figure"

Day#12 of admission

F

F

Objective	
**Vital signs**	RR 15/min, HR 98/min, BP 129/80 mmHg , SpO2 96% on vent
**Patient exam**	Alert, follows simple commands and coughs<br>Lung exam: Bilateral normal sounds.<br>NG tube feeding is very well tolerated.
**Vent alarms**	None
**Diagnostics**	ABG (if learner asks) : 7.42, 39, 104 on ventilator settings of 16/500/70/5<br>CXR (if learner asks): pending

Competencies

**Evaluate patient for weaning on daily basis.**
**Prepare patient before weaning.**
**Interpretation of weaning parameters**
**Watch for post-extubation complications.**
