# Supplementary material for: A Pilot Standardized Simulation-Based Mechanical Ventilation Curriculum Targeting Pulmonary and Critical Care Medicine and Critical Care Medicine Fellows
Source: Avicenna J Med. 2023 Oct 3;13(3):176–81. doi: 10.1055/s-0043-1773792 (PMC10550363; doi:10.1055/s-0043-1773792)
Supplement: Supplementary file 4 — Supplementary Appendix D [file 10-1055-s-0043-1773792-s236d.pdf]

Supplementary Appendix D

Table Mechanical ventilation course overview

Step	Mechanical ventilation course
1	Baseline knowledge test (15 MCQs)
2	A one-on-one high-fidelity case-based simulation session testing MV competencies (34-checklist items) with debriefing
3	A one-hour didactic lecture to the whole group
4	A 45-min hands on session on introduction to knobology and waveforms with RT in small groups of 1–3 fellows
5	A 15-min bedside MV rounds in small groups of 1–3 fellows

Abbreviations: MCQ, multiple-choices questions; MV, mechanical ventilation; RT, respiratory therapist.

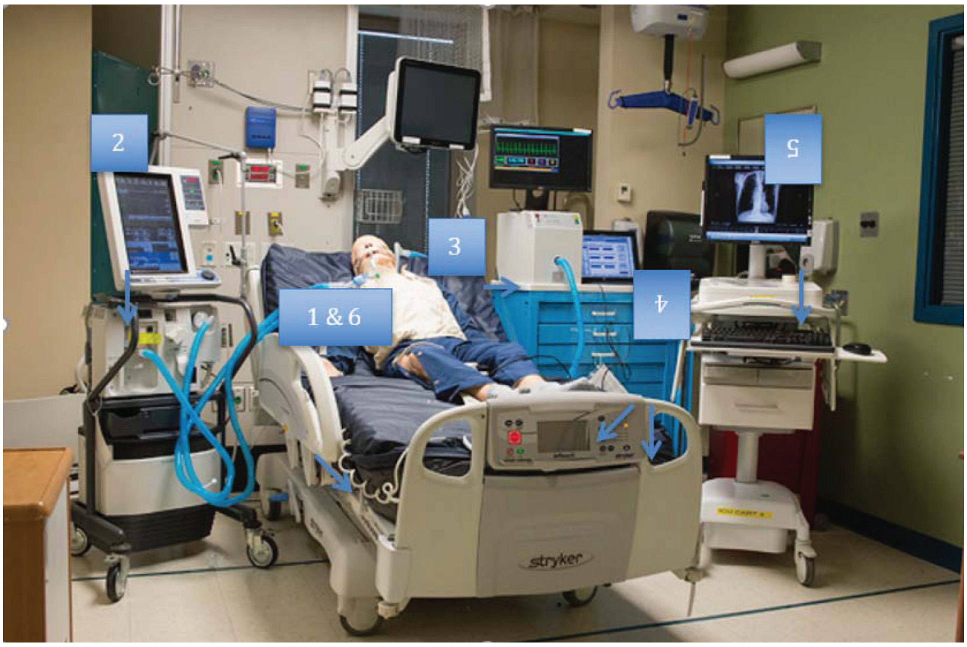

Simulation laboratory at John D. Dingell VA Medical Center, Detroit, MI (author owned)

Equipment and Supply Checklist

1. High-fidelity manikin on a bed or stretcher that can prop head of bed up to 30 degrees.
2. Mechanical ventilator and tubing (must be in a room able to connect to air and oxygen supply).
3. Monitor to display vital signs that connect to the breathing simulator's laptop.
4. ASL 5000 breathing simulator and laptop.
5. Laptop or computer to demonstrate the chest X-ray and scenario steps.
6. Endotracheal tube (size 7.5 or smaller).

The following not shown in the picture but also needed to increase realism:

7. Empty 10 mL syringe with Luer lock (for endotracheal tube cuff inflation/deflation).
8. Bag-valve mask.
7. Non-rebreather mask.
8. Oxygen regulator.

 © 2023. The Author(s).

9. A feeding tube attached to an empty bolus feed bag (placed when the learner asked for it).
10. Venturi mask.
11. Nasal cannula (on extubation).

Train the Trainer

At our site, we trained the clinical educator track fellow to do MV simulation-based teaching for the incoming fellows during the orientation bootcamp. The following schedule is the rough estimate. Master trainers should be prepared to be available from 8 AM to noon on day 1 and 8 AM to 11:30 AM on day 2 of the training. Day 1 is the training-the-trainer. For day 2, a learner will be needed and the new trainer will conduct the teaching under the supervision of the Master Trainer.

Master trainer: Refers to the ICU faculty member responsible for training the clinical educator track fellow and teaching the course.
Trainer: Refers to the clinical educator fellow or other new instructor.
Sample learners: Refers to the 2nd year PCCM fellow recruited for the trainer to practice the MV simulation-based teaching.

Day 1

8:00–8:15 AM—Overview of curriculum to be given to the learners.
8:15–8:30 AM—Review the baseline knowledge/pretest: multiple choice questions.
8:30–11:00 AM—Master trainer runs the simulation case with the new trainer as the learner step-by-step as mentioned in the Supplementary Appendix C. This was followed by principles of debriefing, using scripted and open-ended questions, and the scoring of the 34-checklist items. The master trainer demonstrates how to operate the high-fidelity Laerdal manikin and lung simulator including the trouble shooting of the equipment.
11:00–11:30 AM—Lunch break.
11:30–12:00 PM—Overview of topics to be covered for all other curriculum components.

Day 2

8:00–8:15 AM—Learner takes the baseline knowledge test (15 multiple choice questions).
8:15–9:15 AM—New trainer runs the case simulation with debriefing with the learner, with the master trainer observing and aid in operating the equipment. Prior to debriefing with the learner, the learner will step out, then master trainer and trainer will review together the results of the grading checklist. Then the learner will be called back in for the debriefing. Afterward, the master trainer will review the new trainer's teaching method.
9:15–10:00 AM—Trainer will teach the didactic lecture to the learner.
10:00–10:45 AM—Learner will attend introduction to knobology and waveform session.
10:45–11:00 AM—Learner will attend bedside MV rounds with the trainer.
11:15 AM—Learner completes the posttest multiple-choice questions. Trainer will go over the test with the learner.

 © 2023. The Author(s).
