# Supplementary material for: A Pilot Standardized Simulation-Based Mechanical Ventilation Curriculum Targeting Pulmonary and Critical Care Medicine and Critical Care Medicine Fellows
Source: Avicenna J Med. 2023 Oct 3;13(3):176–81. doi: 10.1055/s-0043-1773792 (PMC10550363; doi:10.1055/s-0043-1773792)
Supplement: Supplementary file 5 — Supplementary Appendix E [file 10-1055-s-0043-1773792-s236e.pdf]

Supplementary Appendix E

Grading Guidelines for the MV Competency Checklist

Total points set to 34

- **Segment A Part 1—Initiating Mechanical Ventilation (total 2 points):**
  1. Learner must make the decision for invasive mechanical ventilation to obtain a point.
  2. Learner must address code status to obtain a point.
- **Segment A Part 2—Initial Ventilator Settings/Postintubation protocols (11 points):**
  3. Correct Tidal Volume (6–8 mL/kg) must be calculated using patient ideal body weight to obtain one point.
  4. Learner must check patient vital signs after intubation to obtain one point.
  5. Intent to order chest X-ray (CXR) must be stated to obtain one point.
  6. Intent to order arterial blood gas (ABG) must be stated to obtain one point.
  7. Learner must assess the patient's need for sedation **and** analgesia (i.e., state intent to order fentanyl) immediately post intubation to obtain one point.
  8. Learner must ask to follow-up on ABG result to obtain one point.
  9. Learner must ask to follow-up CXR result to obtain one point.
  10. Must state need or demonstrate elevating head of bed to at least 30 degrees post intubation to obtain one point.
  11. Must state to initiate chlorhexidine mouthwash post intubation to obtain one point.
  12. Must state to initiate venous thromboembolism (enoxaparin or heparin) and gastrointestinal (famotidine or pantoprazole) prophylaxis to obtain one point
- **Segment B—Auto-PEEP Assessment (2 points):**
  13. Must recognize airway pressure alarm, perform or request expiratory pause while viewing a scalar waveform, and disconnect circuit for a few seconds to obtain one point.
  14. Must initiate appropriate changes to the ventilator to prevent dynamic hyperinflation and Auto-peep. Must get **all** to obtain one point.
  ■ Lower respiratory rate.
  ■ Lower tidal volume
  ■ Decrease inspiration time (Ti)
  ■ Use bronchodilators
  ■ Increase sedation
- **Segment C—Mucous plug (2 points):**
  15. Must recognize/verbalize high peak pressures **and** check plateau pressure using inspiratory pause to obtain one point.
  16. Upon viewing the chest X-ray, must state a differential diagnosis that includes mucous plug and recommend appropriate intervention for a point. Must give **all** therapies for a point:
  ■ Chest physiotherapy + suctioning + bronchodilators, possible bronchoscopy
- **Segment D: Lung protective ventilation in ARDS (1 point)**
  17. Must use lung protective strategy in ARDS. State **all** the below for full points.
  ■ Reduce tidal volume to 6 mL/kg of PBW
  ■ Increase respiratory rate to maintain the minute ventilation
  ■ Check plateau pressures (per ARDSnet guidelines)
  ■ Use PEEP tables to titrate the pressure (tables provided upon request)
- **Segment E: Ventilator Dyssynchrony (2 points):**
  18. Must identify double trigger/Stacking and other types of desynchrony when prompted. Must get **all** for a point.
  ■ Flow hunger
  ■ Double trigger
  ■ Missing trigger

  19. Must describe appropriate intervention. Must get **all** for a point.
  ■ Change flow/Tidal volume (increase for double trigger or flow hunger)
  ■ Change triggers (for missing trigger and double trigger)

 © 2023. The Author(s).

- Change ventilator mode (for all dyssynchrony)
- Increase sedation (for double trigger)

- **Segment F: Liberation/weaning from mechanical ventilation. Exact criterion is per local institution protocol-policies (14 points):**

20. Must examine patient for **weaning/check for resolution** of initial reason for invasive mechanical ventilation (i.e., resolved hypoxia) to obtain one point.
21. Assess **hemodynamics** and stable Vitals to obtain one point.
22. Review **ABG prior** to extubation to obtain one point.
23. Evaluate Ventilator settings, i.e., Low **FiO_2 and PEEP** for one point.
24. Evaluate patient **secretion and** suctioning requirement to evaluate ability of airway protection to obtain one point.
25. Must assess **awakening** trial/ask for sedation interruption to obtain one point.
26. Evaluate patient's neurological status and alertness by asking patient to follow commands and cough to obtain one point.
27. Must discontinue **tube feeding** prior to extubation to obtain one point.
28. Must raise **head of the bed** prior to extubation to obtain one point.
29. Learner must state to initiate spontaneous breathing trial (**SBT**) **and** identify correct duration (minimum of 30 minutes and no longer than 120 minutes) to obtain one point.
30. Must verbalize correct Rapid Shallow Breathing Index (**RSBI = RR/VT(L)**) target (less than 105) and evaluate vitals during SBT to obtain one point.
31. State need or perform a **cuff leak test** before extubation to obtain one point.
32. State correct cuff leak test techniques **either** by deflating ETT balloon and monitoring tidal volume in assist control/volume control setting **or** deflating balloon and listening for audible breath sounds outside of ETT to obtain one point.
33. Must know indications for oxygen supplementation vs non-invasive ventilation post-extubation to obtain one point.
34. Assess patient for stridor/distress and state need to monitor in ICU for overnight or 24 hours after liberation from mechanical ventilation to obtain one point.

 © 2023. The Author(s).
